# A 16-amino acid peptide delays the progression of motor neuron degeneration and pathogenic symptoms in ALS models

**DOI:** 10.1016/j.neurot.2025.e00806

**Published:** 2025-11-26

**Authors:** Cheng-Yung Lin, Bing-Chang Lee, Po-Hsiang Zhang, Shao-Chi Lu, Wei-Zen Chang, Chia-Chuan Wang, Huai-Jen Tsai

**Affiliations:** a Institute of Biomedical Sciences, MacKay Medical University, New Taipei City, Taiwan; b Department of Life Science, Fu Jen Catholic University, New Taipei City, Taiwan; c Institute of Molecular and Cellular Biology, National Taiwan University, Taipei, Taiwan; d School of Medicine, Fu Jen Catholic University, New Taipei City, Taiwan

**Keywords:** Amyotrophic lateral sclerosis, Phosphoglycerate kinase 1, Motor neuron, Zebrafish, SOD1-G93A mice

## Abstract

Amyotrophic lateral sclerosis (ALS) is a progressive motor neurons (MNs) degenerative disease. Despite advancements in understanding ALS pathogenesis, drug development lags far behind. The reduced secretion of phosphoglycerate kinase 1 (Pgk1) by NogoA-overexpressing muscle cells inhibits neurite outgrowth of MNs (NOMNs). However, administration of extracellular Pgk1 (ePgk1) reduces phospho-Cofilin (p-Cofilin), a growth cone collapse marker, and mitigates MN degeneration. This improves NOMNs in NSC34 neural cells and locomotion in SOD1-G93A ALS-mice by suppressing the p-P38-T180/p-MK2-T334/p-Limk1-S323/p-Cofilin-S3 signaling pathway. Here, we identified two Pgk1-based 16-amino acid (aa) short peptides, FD-1 and FD-2, with neuroprotective effects equivalent to those of full-length ePgk1. Administration of FD-1 or FD-2 (FD-1/-2) reduced p-Cofilin and promoted NOMNs in NSC34 ​cells cultured in conditioned medium obtained from NogoA-overexpressing muscle cells. Furthermore, we found that exogenous addition of FD-1/-2 to the culture medium attenuated the accumulation of phospho-Tau-S396 and the cytoplasmic mislocalization of transactive response DNA binding protein of 43 ​kDa (TDP-43) in oxidative-stressed ALS-like SOD1-G93A NSC34 ​cells. In FD-1/-2-injected zebrafish embryos, we observed increased caudal primary MNs branching. In *C9orf72*-knockdown and *hTDP-43-G348C* mRNA overexpressing zebrafish embryos injected with FD-1/-2, axonal growth and motor function were rescued. Moreover, intravenous injection of FD-1/-2 in SOD1-G93A ALS-mice delayed denervation of neuromuscular junction, preserved cell bodies of MNs in the ventral horn of spinal cord, increased grip strength, improved locomotion and prolonged survival. Therefore, both 16-aa short FD peptides are functionally equivalent to full-length 417-aa ePgk1 and thus promising therapeutic short peptides for the treatment of ALS.

## Introduction

Amyotrophic lateral sclerosis (ALS) is characterized by progressive degeneration of motor neurons (MNs) in the brain and spinal cord. This leads to widespread muscle atrophy and paralysis, including the diaphragm muscles, eventually causing respiratory failure [1]. Approximately 10 ​% of ALS cases are familial ALS (FALS), while the remaining 90 ​% are sporadic ALS (SALS). Onset most commonly occurs around 50 years of age with survival expectancy from 3 to 5 years [2]. Many genes associated with ALS pathogenesis have been identified. For example, genetic mutations in the *superoxide dismutase 1* (*SOD1*) gene were the first to be linked to ALS development [3]. Since then, mutations in at least 25 genes have been linked to FALS, SALS, or both. Notable examples include mutations in *Chromosome 9 open reading frame 72* (*C9orf72*) and *Transactive response DNA binding protein of* 43 ​kDa (*TDP-43*)*,* as well as *Fused in Sarcoma* (*FUS*) and *Angiogenin* (*ANG*) [4,5]. However, only around 30 ​% of ALS cases have been attributed to genetic mutations. Therefore, the underlying etiology of most ALS cases remains to be investigated.

The pathogenesis of ALS is often the result of multiple complex interactions. Mutations in *SOD1, TDP43* and *FUS*, for example, can lead to mitochondrial abnormalities and dysfunction, in turn leading to energy deficit in MNs. Mutations in *TDP43* cause nucleocytoplasmic transport dysfunction. Its cytoplasmic accumulation is toxic and also results in MNs degeneration [6,7]. Even abnormal transcellular signaling of non-neuronal cell types are involved in ALS progression. For instance, reactive astrocytes release inflammatory cytokines responsible for neuron death [7,8]. Such causal heterogeneity and complex genetic etiology associated with ALS are roadblocks to drug development and treatment. Therefore, developing drugs for broad application with significant therapeutic benefits remains a critical priority.

Clinical studies of ALS revealed that patients diagnosed with lower motor neuron syndrome and a high level of NogoA detected in muscle cells have a significantly high (88 ​% of 17 examined patients) probability of later progression to full-blown ALS [9]. Additionally, in patients with ALS, overexpression of NogoA in muscles is positively correlated with the functional loss of motor endplates, essential for muscle contraction. Increasing expression of NogoA in muscle cells leads to increasingly severe symptoms [10]. Overexpression of NogoA was also observed in the muscle cells of SOD1-G86R mice, a mouse ALS model [11,12], leading to morphological changes at the neuromuscular junction (NMJ) where MNs communicate with muscle [13]. Meanwhile, overexpressing Rtn4al, the human homolog of NogoA, in muscle cells of adult zebrafish derived from transgenic line *Tg(Zα:TetON-Rtn4al)* could also induce ALS-like phenotypes [14].

Previously, Lin et al. reported a significant reduction of secreted phosphoglycerate kinase 1 (Pgk1) in both NogoA-overexpressing Sol8 muscle cells, a myogenic model, and SOD1-G93A ALS-mice [15]. Meanwhile, they also found that extracellular Pgk1 (ePgk1) could increase the neurite outgrowth of motor neurons (NOMNs) in two cases. First, NOMNs was improved when recombinant Pgk1 was cultured with ALS-mimicking NSC34 ​cells modified to express a mutant human SOD1 protein with a specific G93A mutation (NSC34 SOD1-G93A). Second, NOMNs was also improved when recombinant Pgk1 was cultured with iPSC-derived MNs from a SOD1-G86R patient with ALS. These two outcomes support a non-canonical, glycolysis-independent role for ePgk1 in rescuing ALS-like MNs defects [15]. Moreover, injection of recombinant Pgk1-flag into either the dorsal muscle of ALS-like zebrafish embryos from transgenic line *Tg(Zα:TetON-Rtn4al)* or the gastrocnemius muscle of SOD1-G93A ALS-mice delayed NMJ denervation and improved motor functions [15]. Central to this study, they also determined that ePgk1 caused a decrease in the protein level of p-Cofilin, a growth cone collapse marker, by suppressing the signaling pathway through a mechanism independent of the canonical inhibitory Nogo66/NgR axis [15]. Supplementation of recombinant Pgk1 triggered this cascade and quantifiably improved NOMNs in NSC34 ​cells and locomotion activity in ALS-mice [15]. Fu et al. expanded these studies and discovered that ePgk1 secreted from skeletal muscle cells serves as a ligand that interacts with the neural membranous protein Enolase 2 (Eno2) on motor neurons [16]. In short, they found that interaction between ePgk1 and Eno2 (ePgk1-Eno2) reduced p-Cofilin by repressing the p-P38-T180/p-Limk1-S323/p-Cofilin-S3 axis, thereby enhancing NOMNs *in vitro* and axonal growth *in vivo* [15,16].

Cofilin is a modulator of growth or inhibition. In its phosphorylated state, Cofilin inhibits axonal growth by reducing the rate of actin turnover, which is otherwise essential for dynamic axonal growth. In this work, the reduced expression of p-Cofilin, or dephosphorylated Cofilin, is shown to promote the rapid turnover of actin cytoskeleton, which favors NOMNs.

In more detail, the expression of Cofilin is tightly controlled by two different pathways studied in the present work. First, the Nogo-66 domain of NogoA inhibits axonal growth by binding to the NgR1 receptor and activating the well-established RhoA/ROCK2/p-Limk1-T508/p-Cofilin-S3 pathway [17,18]. So, this cascade causes the upregulation of p-Cofilin and, thus, reduced actin dynamics, axonal growth inhibition and synaptic destabilization [19]. Second, we found that ePgk1-Eno2 interaction suppresses the Rac1/p-Pak1/p-P38/p-MK2/p-Limk1-S323/p-Cofilin-S3 signaling pathway. This cascade causes the downregulation of p-Cofilin, thereby favoring actin dynamics that improve axonal growth and permit synaptic stabilization.

To further elucidate the distinction between the two signaling pathways that control p-Cofilin, we note that p-Limk1-T508, an essential regulator of actin cytoskeleton, regulates actin by phosphorylating and inactivating Cofilin and, as such, promotes cytoskeletal stability and growth inhibition. Interestingly, we demonstrated that the phosphorylation level of p-Limk1-T508, a downstream effector of the Nogo66/NgR1/RhoA/ROCK2/p-Limk1-T508 pathway [18], remains unchanged during ePgk1-Eno2 interaction [15,16]. Instead, we are the first to demonstrate that reducing p-Cofilin expression in NSC34 neural cells is primarily dependent on repressing p-Limk1-S323 through the p-Pak1/p-P38/p-MK2/p-Limk1-S323 axis associated with neurite outgrowth [15,16].

Recently, Lin et al. demonstrated that ePgk1 could prevent dopaminergic neurons from damage caused by the neurotoxin MPTP (1-methyl-4-phenyl-1,2,3,6-tetrahydropyridine). This resulted in improving motor function in zebrafish larvae and adults with Parkinson's disease-like symptoms [20]. This line of evidence suggests that ePgk1 could serve as both neuroprotective and neurotrophic factor. Consequently, ePgk1 is a promising therapeutic protein drug for the treatment of ALS and, potentially, other neurodegenerative diseases.

Administration of ePgk1 could delay NMJ denervation and improve the locomotion of ALS model animals. However, Pgk1 protein is still composed of 417 amino acid (aa) residues with a relatively high molecular weight of 48 ​kDa. This makes its large-scale pharmaceutical preparation costly and impractical. Therefore, in this study, we identified two Pgk1-derived 16-aa short peptides that contain the key functional motifs of wild-type Pgk1. We drew this conclusion from intravenously injecting either full-length 417-aa Pgk1 protein or each Pgk1-derived short FD peptide into the tail vein of SOD1-G93A ALS-mice. We found that the short FD peptides delivered results comparable to those of full-length ePgk1 by preserving NMJ innervation, maintaining grip strength, improving mobility, and prolonging lifespan. This line of evidence suggests that FD-1 and FD-2 are two promising therapeutic short peptides for the treatment of ALS disease.

## Materials and methods

### Ethics approval, consent to participate, and animal housing and care

All experimental animals (mouse and zebrafish) used in this study complied with the protocols of the Institutional Animal Care and Use Committee at Fu Jen Catholic University and MacKay Medical University. All animals in this study were housed in facilities with controlled temperature and humidity. (1) Zebrafish were cultured at 28 ​± ​2 ​°C with a relative humidity of 55 ​± ​10 ​% under a 14-h light/10-h dark cycle. Zebrafish (*Danio rerio*) wild-type AB strain (RRID: ZIRC_ZL1) and transgenic line *Tg(mnx1:GFP)* (RRID: ZIRC_ZL1163) were purchased from ZIRC. (2) Mice were maintained in a controlled environment at 21 ​± ​2 ​°C with a relative humidity of 55 ​± ​10 ​% under a 12-h light/12-h dark cycle. Mouse strains SJL/J (RRID: IMSR_JAX:000686) and B6SJL-Tg(SOD1 G93A)1Gur/J (RRID: IMSR_JAX:002726) were purchased from The Jackson Laboratory, USA. C57BL/6 mice were purchased from the National Center for Biomodels, Taiwan. Strains C57BL/6 and SJL/J were crossbred to generate B6SJL F1 mice which were, in turn, further crossed with B6SJL-*Tg(SOD1 G93A)*1Gur/J to generate ALS-mice used for this study. Before beginning the experiment, all mice underwent genotyping to ensure that each sample contained the pathogenic SOD1-G93A mutation. Then, for each litter, typically 3–5 pups carrying the mutation were identified and randomly dispatched to different treatment groups to avoid littermate bias.

Treatment of ALS-mice followed the Animal Care Guidelines which state that the experimental endpoint is determined by independent animal facility staff members who are not involved in the experimental procedures. Thus, daily monitoring and care of ALS-mice after delivery from the National Center for Biomodels, Taiwan, to FJU were carried out by staff members of the Laboratory Animal Center, FJU Medical School, who were blinded to specific treatments administered to mice in each cage.

### Cell lines and CM collected from culturing Sol8 and Sol8-NogoA cells

NSC34 [21], NSC34-SOD1 G93A [22], Sol8 (ATCC, CRL-2174), and Sol8-NogoA cell lines were maintained in DMEM culture supplemented with 10 ​% FBS, 100 U/mL penicillin, and 0.1 ​mg/mL streptomycin, as previously described by Lin et al. [15]. Cells were cultured in a humidified incubator at 37 ​°C with 5 ​% CO_2_. Sol8 and Sol8-NogoA cells were seeded at a density of 1 ​× ​10^6^ ​cells per well in a 60.8 ​cm^2^ dish in DMEM supplemented with 10 ​% FBS, 100 U/mL penicillin and 0.1 ​mg/mL streptomycin. After culturing for 24 ​h, the medium was replaced by DMEM containing 2 ​% FBS, 100 U/mL penicillin, 0.1 ​mg/mL streptomycin and 1 ​μg/mL doxycycline. After culturing for another 24 ​h, the conditioned medium was collected.

### Recombinant human Pgk1-flag, truncated forms, and short peptides

The cDNAs encoding human Pgk1 and its various truncated forms fused with flag reporter were inserted into pVL1939 vector to generate pVL1939-hPgk1-flag, pVL1939-hPgk1-1/145-flag, pVL1939-hPgk1-146/417-flag, pVL1939-hPgk1-225/417-flag, pVL1939-hPgk1-325/417-flag and pVL1939-hPgk1-1/324-flag. The purification of full-length human Pgk1-flag and its truncated forms was previously described by Lin et al. and Fu et al. [15,16]. After the short peptides were synthesized by Mission Biotech Co., Taiwan, they were dissolved in PBS and stored at −80 ​°C.

### Western blot analysis

The procedure for Western blot analysis was previously described by Lin et al. [15], except the following antibodies were used: anti-Cofilin (RRID: AB_10622000; 1:1000), anti-phospho-Cofilin at S3 (RRID: AB_2080597; 1:1000), anti-α-tubulin (RRID: AB_477579; 1:5000), LIM domain kinase 1 (Limk1) (RRID: AB_648350; 1:1000), phosphorylated Limk1 at S323 (RRID: AB_2136940; 1:1000), phosphorylated Limk1 at T508 (RRID: AB_2136943; 1:1000), P38 mitogen-activated protein kinases (P38) (RRID: AB_330713; 1:1000), phosphorylated P38 at T180 (RRID: AB_331641; 1:1000), chicken anti-goat-HRP (RRID: AB_639230; 1:5000), goat anti-mouse HRP (RRID: AB_955439; 1:5000) and goat anti-rabbit HRP (RRID: AB_2099233; 1:5000). Imaging was performed using the ChemiDoc-It 815 Imaging System.

The inconsistency of band intensity was caused by slight differences in cell culturing conditions among experiments. Therefore, each blot in our experiment was normalized by setting the designated reference group within that blot to 1. This allowed the comparison of band intensity, as shown on Western blot. Values of the other groups in the same blot were then normalized relative to this reference. Consequently, the reference group appears as 1 in each of the three independent experiments, while the other groups are presented as mean ​± ​SD after normalization.

### NOMNs derived from NSC34 ​cells

The *in vitro* study of NOMNs derived from NSC34 ​cells was previously described by Lin et al. [15]. Briefly, NSC34 ​cells were seeded at a density of 3 ​× ​10^3^ ​cells per well in a 9.6-cm^2^ dish in DMEM supplemented with 10 ​% FBS, 100 U/mL penicillin, and 0.1 ​mg/mL streptomycin. After 24 ​h, the medium was replaced with DMEM supplemented with 2 ​% FBS, 100 U/mL penicillin, and 0.1 ​mg/mL streptomycin. After another 24-h incubation, the medium was replaced by conditioned medium collected from culturing Sol8/Sol8-NogoA and supplemented with recombinant Pgk1-flag or Pgk1-derived peptide. Cells were cultured for an additional 48 ​h with a daily change of medium. Images were captured using a Zeiss Axio Observer Z1 microscope, and NOMNs was analyzed with ImageJ software. For each group, a minimum of 90 ​cells were analyzed and averaged from three independent cultures conducted at different time points.

### Ethacrynic acid (EA)-induced oxidative stress

NSC34 ​cells and SOD1-G93A NSC34 ​cells were seeded at a density of 1.3 ​× ​10^5^ ​cells per well in a 9.6 ​cm^2^ dish. They were then differentiated in DMEM supplemented with 2 ​% FBS, 100 U/mL penicillin, and 0.1 ​mg/mL streptomycin, followed by culture over a 72-h period. A concentration of 99 ​ng/mL Pgk1-flag or peptide was added between 24 and 72 ​h of culture time. EA is known to trigger oxidative stress and mitochondrial damage, leading to increased cellular vulnerability and death [23]. Therefore, we induced oxidative stress by adding 40 ​μM ​EA and incubating for 4 ​h before immunohistochemistry and total protein extraction.

### Immunostaining of phosphorylated Tau-S396 (p-Tau-S396) and TDP-43

Antibodies used to perform Western blot analysis were as follows: anti-Tau (RRID: AB_2536235; 1:1000), anti-phospho-Tau at Ser396 (RRID: AB_10860822; 1:1000), anti-α-tubulin (RRID: AB_477579; 1:5000), goat anti-mouse HRP (RRID: AB_955439; 1:5000), and goat anti-rabbit HRP (RRID: AB_2099233; 1:5000). Antibodies used to perform cell immunostaining were as follows: anti-TDP-43 (RRID: AB_615042; 1:200) and Alexa Fluor 594 (RRID: AB_2535860; 1:250). Nuclei were counterstained with 4′,6-diamidino-2-phenylindole (DAPI) (Sigma D9542).

### Caudal primary MNs (CaPMN) branching and axonal growth (AG) observed in zebrafish embryos

The number of branched CaPMNs was examined in zebrafish transgenic line *Tg(mnx1:GFP)* embryos after intracerebroventricular (ICV) injection of Pgk1 and each short FD peptide as 10 ​ng dissolved in 2.3 ​pL of PBS at 20 ​h post-fertilization (hpf). Besides two short FD peptides, we also designed a 16-aa control peptide (MSLSNKLTLDKLDVKG) derived from the 1st to 16th aa residues of Pgk1. This was done to determine whether the branched CaPMNs would be affected by the addition of an arbitrary 16-aa segment. Quantification of the percentage of zebrafish embryos exhibiting branched CaPMNs between the 11th and 20th somites among 30 embryos was performed at 30 hpf. Zebrafish embryos were originated from 60 breeding pairs.

Next, we studied the AG of zebrafish embryos from transgenic line *Tg(mnx1:GFP)*. We performed ICV injection of PBS (control), Pgk1 or each short FD peptide at 20 hpf; this, however, was preceded by microinjection of *C9orf72*-MO or human *TDP43-G348C* mRNA (*hTDP43-G348C* mRNA) at one-cell stage. Then, we observed embryos under confocal microscopy. We then employed cellSens Standard software to measure the lengths of AG presented from the 11th to 20th consecutive somites. AG length (in μm) was averaged from five consecutive axons per embryo, and data were collected from 15 zebrafish embryos per treatment group.

### Tracking the swimming trajectory of zebrafish larvae

Zebrafish embryos untreated and microinjected with *C9orf72* antisense oligonucleotide morpholino (MO) (sequence: TTGTAACATCCATCTGCTGCT- GCAT) (Gene-Tools Inc.) or *hTDP43-G348C* mRNA (50 pg/embryo) were individually placed in 3-cm diameter agar dishes containing 3 ​mL of water. Larvae at 72 hpf were gently touched at the tail part with a fishing line to trigger short-range swimming. A second touch was continuously performed when larvae stopped moving temporally. Three consecutive touches were carried out for each larva. The trajectory of each larva and its total swimming distance (in cm) from three consecutive touches were analyzed using EthoVision XT 17.5 software.

### Intravenous injection in ALS-mice

Mice were anesthetized with Attane (isoflurane) and placed on a heating blanket to maintain body temperature at 37 ​°C. We previously found that an effective dose of exogenous Pgk1-flag (1.5 ​mg/kg) injected biweekly into the gastrocnemius muscle would be sufficient to improve the contractile capability of ALS-mice exhibiting paralyzed leg [15]. Therefore, based on the dose of exogenous Pgk1-flag noted above, we extrapolated the initial dose of Pgk1-flag employed in this study for estimated total skeletal muscle. Such extrapolation yielded an effective dose of 25 ​mg/kg for the whole-body of mouse injected intravenously biweekly. To estimate the dose for short FD peptides, we considered the short half-life of peptides, rather than the entire protein. Accordingly, we modified the regimen by halving the original biweekly dose of Pgk1-flag and administering short peptides FD-1 and FD-2 weekly with a dose of 12.5 ​mg/kg. Therefore, recombinant Pgk1-flag (25 ​mg/kg in 100 ​μL) and short peptides (12.5 ​mg/kg in 100 ​μL) were separately injected biweekly and weekly, respectively, through the tail vein. Intravenous injection was performed at 60 postnatal days (pnd) of each mouse until reaching humane endpoint. Endpoint is characterized by the development of limb paralysis when mice cannot use their forelimbs to grasp food for self-feeding. The number of survival days before each mouse reached endpoint was recorded and analyzed using accumulative Kaplan-Meier survival curves.

### NMJ denervation

We also determined if administration of short peptides could delay disease progression. To accomplish this, we first intravenously injected 60-pnd-old SOD1-G93A ALS-mice with short FD peptide and then dissected the gastrocnemius muscles from the same mice at 75 postnatal days. To study the denervation of NMJ, we followed the immunostaining protocol described by Lin et al. [15]. Frozen sections of mouse gastrocnemius muscle were prepared using a Leica CM3050S cryostat set to OT -23 ​°C and CT -20 ​°C with a section thickness of 10 ​μm. The following antibodies were used to label presynaptic and postsynaptic components of the NMJ: anti-Synapsin 1 (Syn1) (RRID: AB_2616578; 1:100), Rabbit IgG CY2-conjugated, pre-adsorbed secondary antibody (RRID: AB_218573; 1:250), and CF®568-conjugated α-bungarotoxin (BTX; 00006–100 ​μg; 1:100). Confocal images of Syn1 and α-BTX colocalized signals were acquired using a Zeiss LSM 780 confocal microscope. Signal colocalization was quantified by MetaMorph software as the ratio of Syn1 and α-BTX overlapping puncta to total α-BTX-positive puncta within a 277.5 ​μm^2^ region (400 ​× ​400 pixels) and expressed as a percentage. For each sample, colocalization percentage was calculated as the average taken from four randomly selected muscle regions. Data of each group were averaged from three independent samples.

### Collection of mouse spinal cord

The collection of mouse spinal cord followed the procedures described by Kennedy et al. [24] with some modifications. Adult mice were anesthetized by intraperitoneal injection of sodium pentobarbital. Then, the chest cavity was opened, and mice were euthanized through whole-body transcardial perfusion with PBS, followed by perfusion with 4 ​% paraformaldehyde (PFA). Next, using surgical scissors and forceps, the skin along the back of the animal's head and spine was removed. The muscles along the entire length of the spine were carefully dissected to expose the vertebrae. A fine-tipped angled bone nipper was used to make a small incision at the lower lumbar region, typically at the level of the hip. The dorsal side of the spinal column was gently opened with the bone nipper by making one or two cuts on either side and removing the resulting bone flap. Any connective tissue obstructing the spinal cord was cleared by using microscissors. Moving from caudal to cranial, dissection continued along the entire length of the spine until the spinal cord was fully exposed, and the L5 segment of the spinal cord was collected. Sample was removed, fixed in 4 ​% PFA for 24 ​h, and then gradually transferred through sucrose gradient solution (10 ​%, 20 ​%, and 30 ​% sucrose) for cryoprotection. Each sucrose concentration was replaced only after the sample had completely settled at the bottom of the container.

### Cell bodies of MNs in the ventral horns of spinal cord

We examined the number of cell bodies (soma) of MNs in the ventral horn of spinal cord at lumbar 5 (L5) in 100-pnd-old SOD1-G93A ALS-mice treated with PBS (negative control), Pgk1 and FD-1/-2 short peptides. B6/SJL (littermate control) mice served as the strain control. Samples were first washed with PBS for 5 ​min to remove any residual debris or fixatives. Subsequently, 200 ​μL of acetone were added to permeate the cell membranes, allowing antibody penetration. The samples were covered with parafilm to prevent acetone evaporation and incubated at 4 ​°C for 10 ​min. Following permeation, the samples were washed again with PBS for 10 ​min to remove excess acetone. To block nonspecific binding, samples were incubated in 5 ​% bovine serum albumin (BSA) in 0.1 ​% Triton X-100/PBS for 1 ​h.

To count the number of MN cell bodies, we performed immunochemical staining using primary antibody anti-choline acetyltransferase (ChAT) (1:100 dilution in 5 ​% BSA, 0.1 ​% Triton X-100/PBS, RRID: AB_2079751). ChAT is a detectable enzyme that produces acetylcholine, a neurotransmitter that regulates muscle movement and other functions, and expresses in the cytosol of MNs. Anti-neuron-specific nuclear protein (NeuN) (1:100 dilution in 5 ​% BSA, 0.1 ​% Triton X-100/PBS, RRID: AB_2736206) was used to detect NeuN in nuclei. Primary antibodies were added to the samples and incubated at 4 ​°C for two days to allow specific binding to their respective target proteins. After incubation, samples were repeatedly washed with PBS for 10 ​min to remove any unbound antibody. Then, secondary antibodies, including Alexa Fluor 488 (1:250 dilution in 5 ​% BSA, 0.3 ​% Triton X-100/PBS, RRID: AB_2534102) and Alexa Fluor 594 (1:250 dilution in 5 ​% BSA, 0.3 ​% Triton X-100/PBS, RRID: AB_2535860), were applied and incubated overnight at 4 ​°C to label primary antibody. After overnight incubation, samples were repeatedly washed with PBS for 5 ​min to ensure removal of excess secondary antibody. Finally, samples were mounted with a mounting solution, sealed with nail polish to secure the coverslips, and stored at 4 ​°C to preserve the fluorescent signals for subsequent imaging. Each group consisted of three mice, and four spinal cord sections were captured from each mouse. Images of both ventral horns were taken using a LEICA TCS SP5 II confocal microscope with 20× magnification objective. The cell bodies of MNs exhibiting a yellow signal (green overlapped with red signals) and cell width greater than 20 ​μm were counted. The number of MNs colabeled with ChAT and NeuN was averaged from four different fields of view and served as the representative value of one sample. Data of each group were averaged from three independent ALS-mice.

### Grip strength test

To assess limb muscle strength in mice, we employed a grip strength meter (GSM, BioSeb Instruments, BIO-GS3, France) equipped with the appropriate grid and T-bar attachments. Prior to performing the trial, mice were arranged for acclimation to the test room overnight, and the meter was reset to zero. Each mouse was held gently by the tail, forepaws positioned near the grip handle, allowed to grip the grid or T-bar naturally, and then pulled backward horizontally until release. The meter recorded maximum force automatically. If the tested mouse became fatigued during testing, we allowed it to rest for 10 ​min, after which we continued with the test. For each mouse, we carried out five trials, and its grip strength was presented as the median value calculated from five repeated measurements. The grip strength test of each mouse was started at 60 days post-birth and examined every five days until each mouse in the control group died.

### Mouse tracking analysis

Mice were raised in 32 ​cm ​x ​15.5 ​cm x 15 ​cm cages. Their locomotive activities were recorded for 5 ​min to obtain trajectories. Using EthoVision XT 17.5, the locomotion distance (in cm) of each mouse during this period of time was measured. Data were averaged from five individual mice.

### Statistical analysis

All data are presented as mean ​± ​SD. The number of biological replicates (N) is indicated in the corresponding figure legends. All *in vivo* data were derived from independent biological samples. *In vitro* experiments were conducted across at least three separate differentiation batches. For comparisons involving a single control group, parametric data were analyzed using one-way ANOVA followed by Dunnett's multiple comparisons test. Pairwise comparisons were evaluated using a two-tailed, unpaired Student's t-test. The neurite length and the number of MN cell bodies in the ventral horns of spinal cord were analyzed using ANOVA with repeated measures. Survival curves were compared with the log-rank (Mantel-Cox) test. All statistical analyses were performed using GraphPad Prism version 9.

## Results

### Domain mapping of Pgk1 to screen for minimally functional domain able to reduce p-Cofilin

**Fig. 1:**
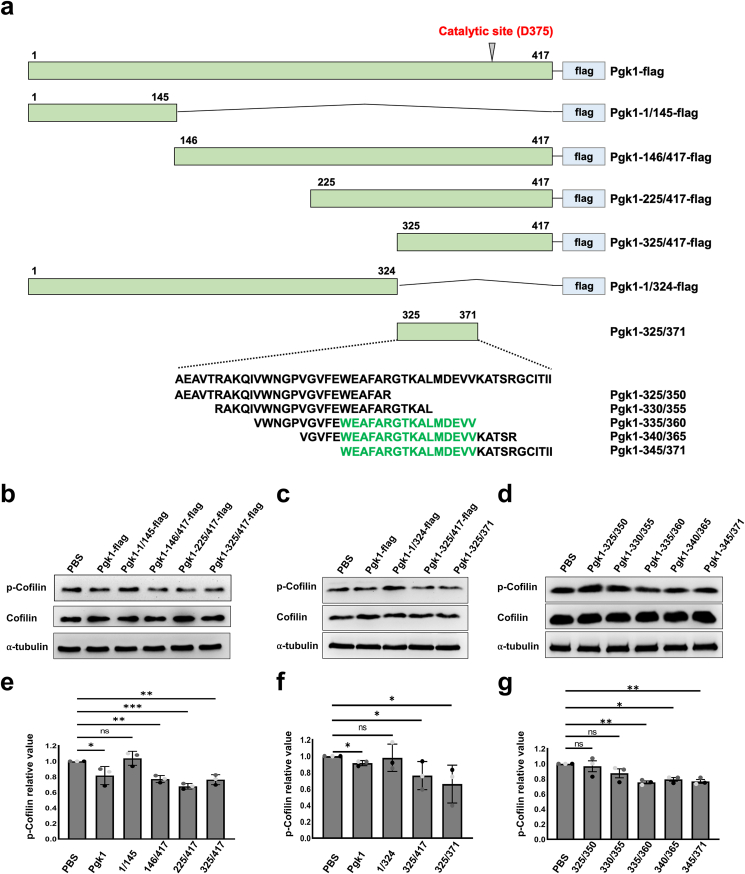
**Identification of the functional domain of Pgk1 that promotes neurite outgrowth of motor neurons.(a)** Schematic diagrams of various truncated forms derived from full-length human Pgk1 (1–417 amino acids) fused with reporter Flag (Pgk1-flag). Each truncated form was indicated by its start and stop amino acid residues, such as Pgk1-1/145-flag. **(b**–**d)** Western blot analysis was used to detect the expressed level of protein. Motor neuron NSC34 ​cells were cultured in Sol8-NogoA CM. The protein levels of p-Cofilin, total Cofilin and α-tubulin, which served as internal loading control, were detected and quantified. The relative value of p-Cofilin was determined as the ratio of p-Cofilin to α-tubulin. The relative value of p-Cofilin in Sol8-NogoA CM added with PBS, which served as the control group, was normalized as 1. Compared to the control group set as 1, the relative value of p-Cofilin obtained from each group, including addition of full-length and truncated forms, or peptides, as indicated, was calculated, respectively. **(e**–**g)** Quantification of the relative p-Cofilin value of each group. Value of each group was averaged from three independent experiments and presented as mean ​± ​SD. Statistical analysis was based on one-way ANOVA (∗, p ​< ​0.01; ∗∗, p ​< ​0.005; ∗∗∗, p ​< ​0.001, ns, no significance).

In NSC34 neural cells cultured in CM obtained from NogoA-overexpressing Sol8 muscle cells (Sol8-NogoA CM) and supplemented with recombinant Pgk1-flag, p-Cofilin is reduced [15]. Here, we generated and then examined various truncated forms of human Pgk1 for their ability to reduce p-Cofilin to favor NOMNs (Fig. 1a). To accomplish this, NSC34 ​cells were cultured in Sol8-NogoA CM and supplemented with different Pgk1-based truncated forms. Compared to the PBS-supplemented control group, p-Cofilin was significantly reduced in NSC34 ​cells cultured with Pgk1-146/417-flag, Pgk1-225/417-flag and Pgk1-325/417-flag (Fig. 1b and e). Meanwhile, p-Cofilin levels remained unchanged in the Pgk1-1/145-flag- and Pgk1-1/324-flag-treated cells (Fig. 1b, c, e and f). These results suggest that the truncated form from the 1st to the 324th amino acid of Pgk1 (segment 1/324) could not be a functional domain. However, segments 146/417 (Fig. 1, b and e), 225/417 (Fig. 1, b and e), 325/417 (Fig. 1, b, c, e and f) and 325/371 (Fig. 1c and f) could all reduce p-Cofilin. Therefore, we deduced that the minimally functional domain involved in improving NOMNs would, most likely, be located within segment 325/371.

Within segment 325/371, cells treated with segments 325/350 and 330/355 were not significantly different from those of PBS-treated controls. Instead, results demonstrated that p-Cofilin expression was reduced in cells treated with segments 335/360, 340/365, and 345/371 (Fig. 1d and g). These findings suggest that supplementation with these three 25-aa peptides might improve NOMNs by suppressing p-Cofilin just like full-length ePgk1. However, since all three segments exhibited equivalent functional efficacy, we next screened for the shortest functional segment that could still improve NOMNs. This was determined to be a 16-aa sequence located within the 345–360 region, which is conserved to all three peptides, and it was designated as FD-1. Apart from wild-type FD-1, we also employed a mutant for parallel studies. Accordingly, we randomly selected a single-aa mutation, FD-2, in which alanine at the 354th residue of FD-1 was replaced by proline to perform a comparative analysis with FD-1.

### Extracellular addition of Pgk1-based short peptides in CM improved NOMNs *in vitro*

**Fig. 2:**
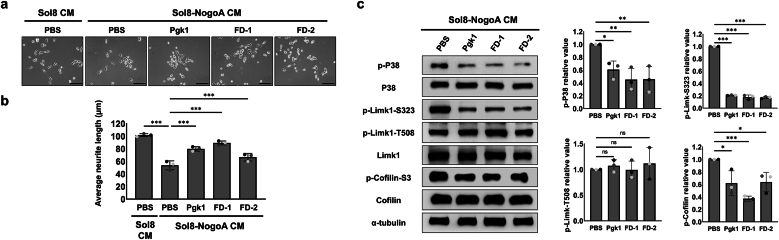
**The effect of supplementing each short FD peptide in CM on the neurite outgrowth of motor neurons.(a)** Neurite growth derived from motor neuron NSC34 ​cells cultured in CM, either from skeletal muscle Sol8 cells (Sol8) or from NogoA-overexpressing Sol8 cells (Sol8-NogoA), with supplementation of PBS (negative control), Pgk1 (positive control) and short peptides (FD-1 and FD-2), as indicated, was observed under confocal microscopy. NSC34 cells cultured in Sol8 CM supplemented with PBS was another control group. Scale bar: 50 ​μm. **(b)** Length of neurite outgrowth derived from NSC34 ​cells. For each group, a minimum of 90 ​cells were randomly selected, analyzed each time, and quantified using ImageJ software. The final data were averaged from three independent trials in which cells were cultured from different batches. Statistical analysis was performed using a mixed-effects model (ANOVA with repeated measures) (∗∗∗, p ​< ​0.001). **(c)** Western blot analysis. The expression levels of p-P38, p-Limk1-S323, pLimk1-T508, total Limk1 (Limk)1, p-Cofilin, total Cofilin (Cofilin) and α-tubulin, which served as loading control, in NSC34 ​cells treated individually with PBS, Pgk1, and peptide, as indicated, were examined. Quantification of the results from Western blotting. The intensity of each band was digitized by ImageJ software. The relative value of p-P38, p-Limk1-S323, p-Limk1-T508 and p-Cofilin was calculated as the ratio of each variable to α-tubulin, respectively. The relative value obtained from the Sol8-NogoA CM-treated PBS control group was normalized as 1. The relative values of p-P38, p-Limk1-S323, p-Limk1-T508 and p-Cofilin resulting from the addition of Pgk1 and two peptides separately were calculated and compared among the three groups based on statistical analysis using one-way ANOVA (∗, p ​< ​0.01; ∗∗, p ​< ​0.005; ∗∗∗, p ​< ​0.001; ns, no significance).

NSC34 ​cells cultured in Sol8 CM and incubated with PBS, which served as control, had an average neurite length of 101.7 ​± ​2.3 ​μm ​(N ​= ​298). However, in PBS-treated NSC34 ​cells cultured in Sol8-NogoA CM, NOMNs was inhibited, showing an average neurite length of 53.6 ​± ​7.2 ​μm ​(N ​= ​436) (Fig. 2a and b). In contrast, averaged neurite lengths of 79.8 ​± ​3.6 ​μm ​(N ​= ​361), 89.2 ​± ​3.1 ​μm ​(N ​= ​370) and 66.6 ​± ​6.0 ​μm ​(N ​= ​439) were observed in NSC34 ​cells cultured in Sol8-NogoA CM supplemented with Pgk1 (positive control), short peptide FD-1 and FD-2, respectively (Fig. 2a and b). From these results, we conclude that the two short FD peptides could rescue the inhibited growth of NOMNs in a manner similar to that of full-length ePgk1.

The level of phosphorylated P38 (p-P38) can interfere with axonal transport with implications in neurological pathogenesis. In addition to p-P38, we also detected the levels of p-Limk1-S323 p-Limk1-T508 and p-Cofilin expressed in NSC34 ​cells. In the supplementary PBS group (control), p-P38, p-Limk1-S323, p-Limk1-T508 and p-Cofilin were all highly expressed in NSC34 ​cells cultured in Sol8-NogoA CM. However, when supplemented with Pgk1, FD-1 or FD-2, NSC34 ​cells cultured in Sol8-NogoA CM exhibited significantly reduced levels of p-P38, p-Limk1-S323, and p-Cofilin (Fig. 2c). Meanwhile, the level of p-Limk1-T508 remained unchanged (Fig. 2c). A reduction in the expression of p-Limk1-S323 leads to decreased p-Cofilin, as noted above, and thus increases NOMNs. This line of evidence suggests that short peptides FD-1 and FD-2 can be expected to play a neuroprotective role similar to that of full-length ePgk1 since they can also suppress the p-P38/p-Limk1-S323/p-Cofilin-S3 axis.

### Addition of either Pgk1 or FD peptides could alleviate oxidative stress-induced p-Tau-S396 accumulation and TDP-43 mislocalization in ALS-like MN cells

**Fig. 3:**
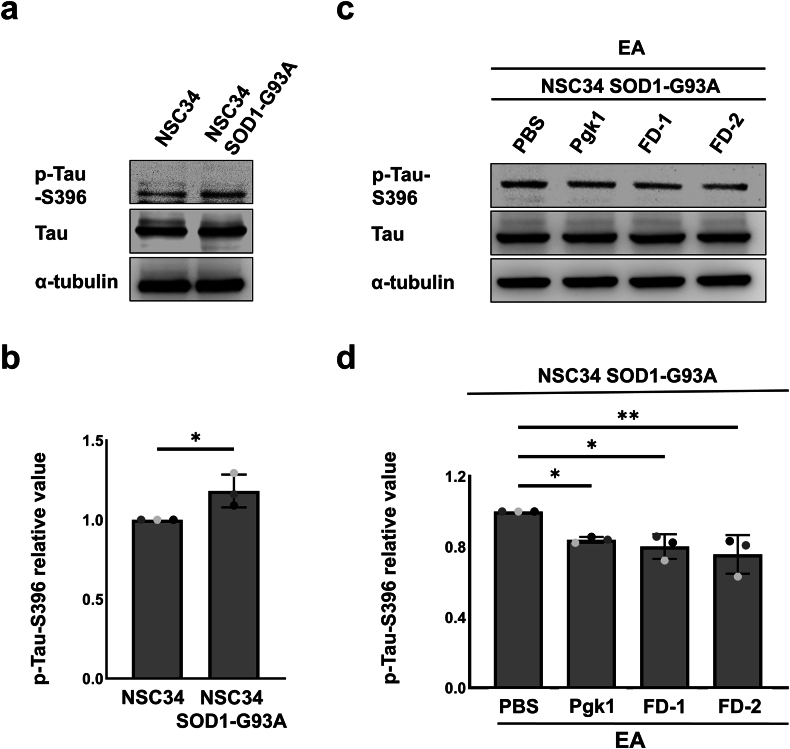
**Addition of extracellular Pgk1 and each short FD peptide could alleviate p-Tau-S396 accumulation in ALS-like motor neuron cells.(a, c)** Using Western blot to analyze the expression levels of p-Tau-S396 in NSC34 ​cells and NSC34-SOD1 G93A cells, as well as NSC34-SOD1 G93A cells treated with EA and added with PBS (EA-treatment control group), Pgk1, FD-1 or FD-2 in medium. **(b, d)** Quantification and statistical analysis of band intensities from Western blotting. The intensity of each band was digitized by ImageJ software. The relative intensity of p-Tau-S396 was calculated as the ratio of p-Tau-S396 intensity to that of total Tau (Tau). **(b)** The relative value obtained from NSC34 was normalized as 1. The relative values of p-Tau-S396 from NSC34 and NSC34-SOD1 G93A cells were compared, and statistical analysis was performed using one-tailed, unpaired Student's t-test. (∗, p ​< ​0.05). **(d)** The relative value obtained from the EA ​+ ​PBS-treated NSC34-SOD1 G93A group was normalized as 1. The relative value of p-Tau-S396 from NSC34-SOD1 G93A cells treated with EA and added with Pgk1, FD-1 or FD-2 was calculated. Data from each group were obtained from three independent experiments labeled with different colors on the plot. The final averaged values were compared among the four groups based on statistical analysis using one-way ANOVA (∗, p ​< ​0.01; ∗∗, p ​< ​0.005).

**Fig. 4:**
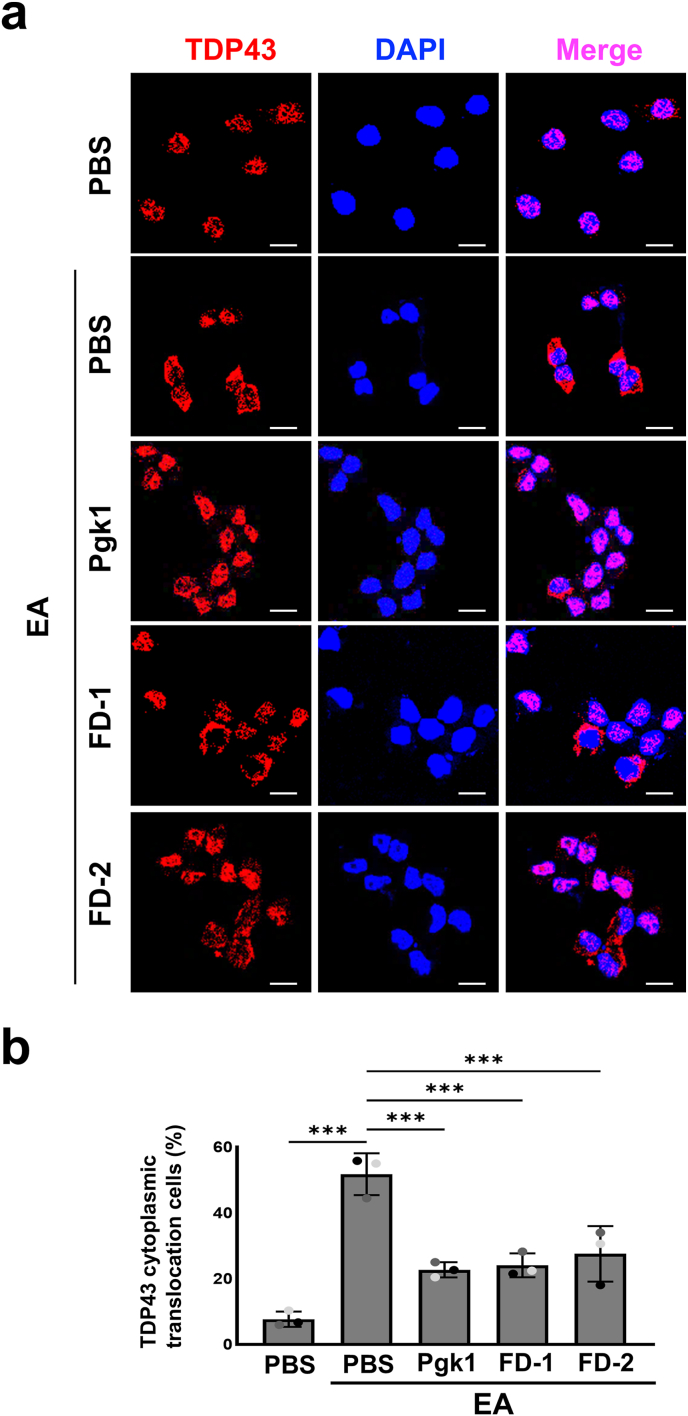
**Addition of Pgk1 and each short FD peptide could alleviate the cytoplasmic mislocalization of TDP-43 in NSC34 neural cells under oxidative stress.(a)** Representative immunofluorescence images showing TDP-43 localization in NSC34 motor neuron cells. TDP-43 (red) and nuclei (DAPI, blue) were visualized, and merged images are shown in pink. Cells were treated with PBS, EA and PBS, EA and Pgk1, EA and FD-1, and EA and FD-2, as indicated. **(b)** The percentage of TDP-43 cytoplasmic mislocalization in MNs. For each group, a minimum of 45 ​cells were randomly selected and analyzed. The final data were averaged from three independent trials in which cells were cultured from different batches. The percentage exhibiting TDP-43 cytoplasmic translocation in MNs from each group was compared. One-way ANOVA was used for statistical analysis (∗∗∗, p ​< ​0.001). Scale bar: 10 ​μm.

It is well known that p-Tau-S396 accumulation in ALS MNs contributes to mitochondrial fragmentation and oxidative stress [25,26]. The results showed that ALS-like SOD1-G93A NSC34 ​cells displayed a higher basal expression level of p-Tau-S396 compared to that of wild-type NSC34 ​cells (Fig. 3a and b). However, p-Tau-S396 expression was effectively reduced in SOD1-G93A NSC34 ​cells treated with EA combined with individual exogenous addition of Pgk1, FD-1 or FD-2 to the culture medium (Fig. 3c and d). Untreated NSC34 ​cells also showed a predominance of TDP-43 in the nucleus with translocation of TDP-43 from nucleus to cytoplasm observed in only 7.6 ​± ​2.3 ​% of cells (Fig. 4a and b). Again, upon treating NSC34 ​cells with EA, translocation of TDP-43 from nucleus to cytoplasm was observed in 51.7 ​± ​6.4 ​% of cells (Fig. 4a and b). However, upon adding Pgk1, FD-1 or FD-2 into the culture medium, the number of EA-treated NSC34 ​cells undergoing cytoplasmic translocation of TDP-43 was proportionately reduced to 22.7 ​± ​2.3 ​%, 24.0 ​± ​3.6 ​% and 27.5 ​± ​8.4 ​%, respectively (Fig. 4a and b). Therefore, similar to the neuroprotective function of full-length of Pgk1, both FD-1 and FD-2 could mitigate oxidative stress-induced p-Tau-S396 accumulation and TDP-43 cytoplasmic mislocalization, potentially alleviating their pathological effects in ALS models.

### Administration of each short FD peptide resulted in increased ectopic axonal branching, improved AG and motor function in zebrafish embryos

**Fig. 5:**
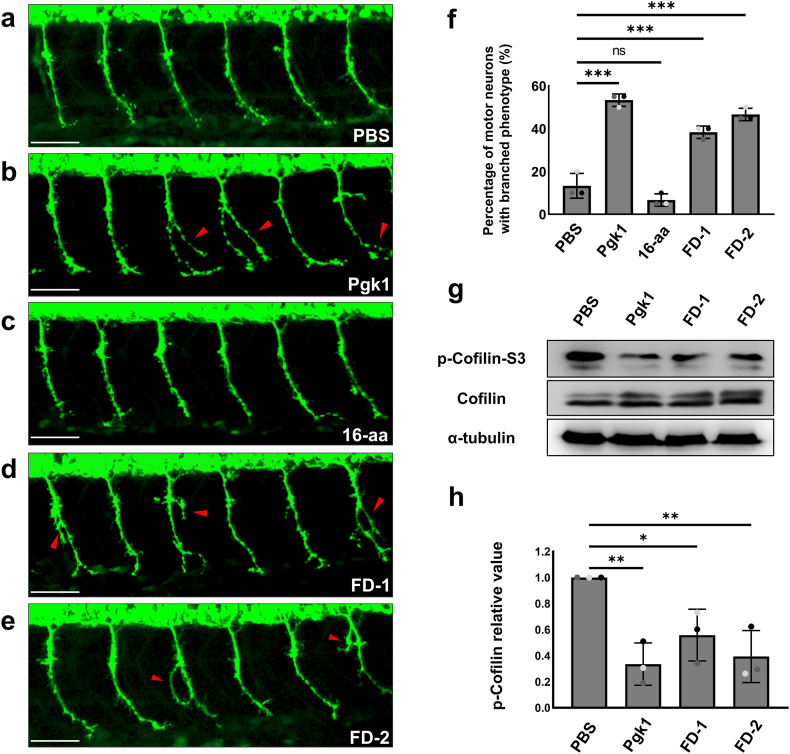
**ICV injection of each short FD peptide increased ectopic axonal branching of motor neurons in zebrafish embryos.(a**–**e)** Motor neurons tagged with green fluorescence in zebrafish embryos from transgenic line *Tg(mnx1:GFP)* were observed under confocal microscopy at 30 hpf following ICV injection of each peptide in embryos at 20 hpf. Branched ectopic axons derived from CaPMNs located in the 11th to 20th somites of embryos were observed. Then, the number of motor neurons exhibiting this ectopic axonal branching, as indicated by red arrowheads, was counted in the following groups: **(a)** PBS group: served as an ICV injection control; **(b)** Pgk1 group: injection of full-length of Pgk1 which served as positive control; **(c)** 16-aa group: injection of an arbitrary non-functional 16-aa peptide (see Materials and Methods) which served as a control group and **(d, e)** short peptide groups: injection of FD-1 and FD-2, respectively. **(f)** The percentage of embryos exhibiting ectopic axonal branching among the total number of examined embryos in each group was quantified. For each trial, the number of branched neurite embryos was averaged from 30 embryos, and the experiment was repeated three times (total N ​= ​30). Data were presented as mean ​± ​SD. One-way ANOVA was used to perform statistical analysis (∗∗∗, p ​< ​0.001; ns, no significance). Scale bar: 50 ​μm. **(g)** Western blot analysis. The expression levels of p-Cofilin (p-Cofilin-S3), total Cofilin (Cofilin) and α-tubulin in zebrafish embryos treated with either PBS, Pgk1, or short peptides, as indicated, were examined. **(h)** Quantification of the results from Western blotting. The intensity of each band was digitized by ImageJ software. The α-tubulin served as an internal loading control. The p-Cofilin level of each treatment relative to its own internal control was calculated. Relative p-Cofilin from the PBS-injected control group was normalized as 1. The change (in fold) of relative intensity of p-Cofilin against α-tubulin was compared to that of the control group which was set as 1. Data were calculated from three independent experiments marked in three different colors and presented as mean ​± ​S.D. (n ​= ​3). The relative values of p-Cofilin resulting from the addition of Pgk1, FD-1 and FD-2 were calculated and compared among the three groups based on statistical analysis using one-way ANOVA (∗, p ​< ​0.01; ∗∗, p ​< ​0.005).

Zebrafish embryos from transgenic line *Tg(mnx1:GFP)* were subjected to ICV injection with Pgk1, FD-1 or FD-2. In PBS-injected control and 16-aa-injected control groups, the percentage of branched CaPMNs was 13.3 ​± ​5.7 ​% and 6.7 ​± ​2.9 ​%, respectively, at 30 hpf (Fig. 5 a, c and f). This result indicated that branched CaPMNs were not increased in the arbitrary 16-aa-injected group, which contains a segment from 1st to 16th amino acid residues of Pgk1. However, the percentages of Pgk1- (Fig. 5b), FD-1- (Fig. 5d) and FD-2-injected (Fig. 5e) zebrafish embryos exhibiting ectopic branched CaPMNs were respectively increased to 53.3 ​± ​2.9 ​%, 38.3 ​± ​2.9 ​% and 46.7 ​± ​2.9 ​% at 30 hpf (Fig. 5f).

Based on the collective evidence, we can infer that supplementary short peptides FD-1 and FD-2 exhibit neuroprotective efficacy equal to that of supplementary full-length ePgk1 by the improvement in branched CaPMNs. We further reasoned that both FD peptides must, therefore, contain an essential domain (345th to 360th segment) responsible for MNs growth, even while FD-2 contains a single-aa mutation at the 354th residue. Meanwhile, it is noteworthy that supplementation of an arbitrary 16-aa peptide could not improve branched CaPMNs. This contrast suggests that the 1st to the 16th amino acid segment of Pgk1 is not responsible for MNs growth. Additionally, we ruled out the possibility that the exogenous addition of an arbitrary 16-aa segment could increase the number of branched CaPMNs.

Compared with the PBS-injected control group, we also observed a decreased level of p-Cofilin in zebrafish embryos injected with exogenous Pgk1, FD-1, or FD-2 (Fig. 5g and h). Therefore, we conclude that ICV administration of short peptides FD-1 and FD-2 could, like ePgk1, significantly reduce p-Cofilin level and, thus, increase axonal branching in zebrafish embryos.

**Fig. 6:**
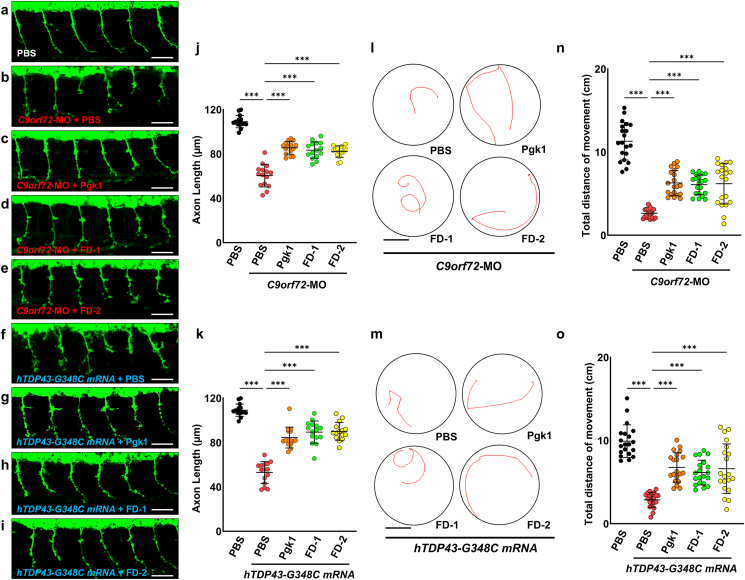
**AG and motor function were improved in ALS-like zebrafish embryos by ICV injection with each short FD peptide.(a**–**i)** Embryos harboring GFP-tagged motor neurons from transgenic line *Tg(mnx1:GFP)* were employed to perform ICV injection of material, as indicated, at 20 hpf. The axonal growth of CaPMNs located between the 11th and 20th somites of embryos at 30 hpf was observed using confocal microscopy. Scale bar: 50 ​μm. **(a)** Injection of PBS served as injection ICV control. **(b**–**e)** Injection of *C9orf72*-MO at one-cell stage, followed by ICV injection of PBS, Pgk1, FD-1 and FD-2. **(f**–**i)** Injection of *hTDP43-G348C* mRNA at one-cell stage, followed by ICV injection of PBS, Pgk1, FD-1, and FD-2. **(j, k)** Quantification of the length of axonal growth of embryos injected with PBS alone and injected with *C9orf72*-MO combined individually with PBS, Pgk1, FD-1 and FD-2 and injected with human *TDP43-G348C* (*hTDP43-G348C)* mRNA combined individually with PBS, Pgk1, FD-1 and FD-2 (N ​= ​15). **(l, m)** Swimming trajectories and **(n, o)** swimming distance of 72-hpf embryos injected with *C9orf72*-MO combined individually with PBS, Pgk1, FD-1 and FD-2 and injected with *hTDP43-G348C* mRNA combined individually with PBS, Pgk1, FD-1 and FD-2 (N ​= ​15). Scale bar: 1 ​cm. Statistical analysis was performed using one-way ANOVA (∗∗∗, p ​< ​0.001).

We also found that zebrafish embryos exhibited normal axonal development of 109.1 ​± ​5.5 ​μm ​at 30 hpf (Fig. 6a and j). Yet, the length of AG was significantly shortened in the *C9orf72-*MO plus PBS-injected embryos at 60.4 ​± ​10.2 ​μm (Fig. 6b and j). Nevertheless, this shortened axonal defect could be rescued by injecting either *C9orf72*-MO plus Pgk1, *C9orf72*-MO plus FD-1, or *C9orf72*-MO plus FD-2. Post-rescue, axonal lengths were measured as 85.5 ​± ​5.7, 83.4 ​± ​7.4 and 82.1 ​± ​5.1 ​μm, respectively (Fig. 6c, d, e and j). These results suggest that administration of FD-1 and FD-2 could improve AG in C9orf72-knockdown embryos in a manner similar to that driven by extracellular addition of Pgk1.

Shortened AG at 53.0 ​± ​9.8 ​μm was similarly observed in embryos injected with *hTDP43-G348C* mRNA plus PBS at 30 hpf (Fig. 6f and k). Nonetheless, the defect was rescued by *hTDP43-G348C* mRNA plus Pgk1 injection, *hTDP43-G348C* mRNA plus FD-1 injection and *hTDP43-G348C* mRNA plus FD-2 injection, as indicated by AG reaching 84.5 ​± ​9.4, 89.4 ​± ​10.0 and 90.0 ​± ​8.0 ​μm, respectively (Fig. 6g, h, i and k). Again, administration of FD-1 and FD-2 could improve AG in C9orf72-knockdown- and hTDP43-G348C-overexpressing embryos in a manner similar to rescue driven by Pgk1.

Zebrafish swimming trajectory and distance can be used to quantify responses to stimuli (Fig. 6l and m). We found the total swimming distance of larvae developed from embryos injected with *C9orf72*-MO plus PBS (Fig. 6n) and *hTDP43-G348C* mRNA plus PBS (Fig. 6o) to be 2.6 ​± ​0.6 ​cm and 2.9 ​± ​0.9 ​cm, respectively. These swimming distances were shorter than that of PBS-injected control. However, this swimming defect was improved in embryos injected with *C9orf72*-MO plus Pgk1, plus FD-1 or plus FD-2 with post-rescue values of 6.3 ​± ​1.5 ​cm, 6.1 ​± ​1.2 ​cm and 6.2 ​± ​2.5 ​cm, respectively (Fig. 6n). In addition, embryos injected with either *hTDP43-G348C* mRNA plus Pgk1, *hTDP43-G348C* mRNA plus FD-1 or FD-2 showed post-rescue values of 6.8 ​± ​1.8 ​cm, 6.1 ​± ​1.5 ​cm and 6.6 ​± ​3.0 ​cm, respectively (Fig. 6o). Collectively, this evidence suggested that short FD peptides, like full-length Pgk1, could also increase locomotive capability in ALS-like transient transgenic zebrafish.

### Denervation in NMJ was decreased in ALS-like mice intravenously injected with each short FD peptide

**Fig. 7:**
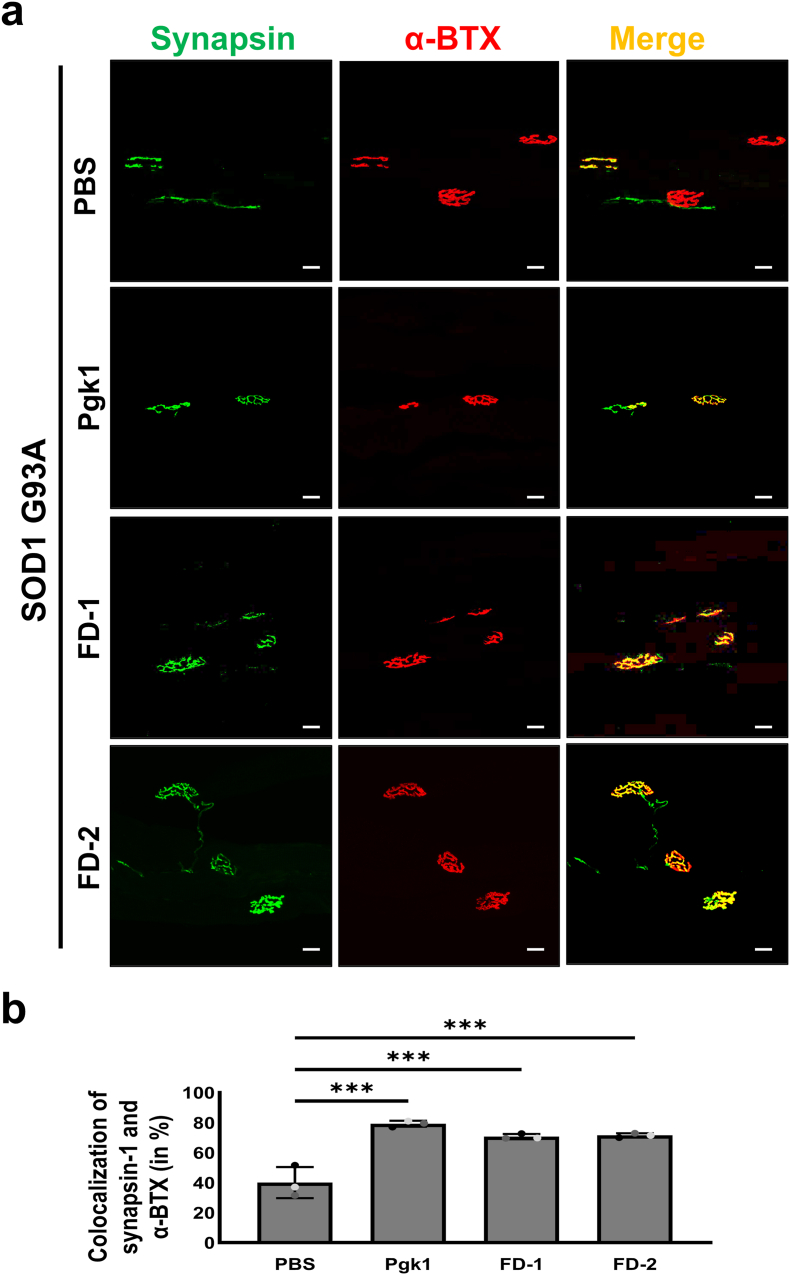
**The denervation that occurred in NMJ of ALS-mice was rescued by intravenous injection with each short FD peptide.(a)** Images were observed under confocal microscopy after dissection of gastrocnemius muscles from the hind leg of SOD1 G93A ALS-mice at 75 days after birth, followed by immunohistochemical staining. ALS-mice 60 days after birth were individually treated with PBS (N ​= ​3, 2♂1♀), Pgk1 (N ​= ​3, 1♂2♀), FD-1 (N ​= ​3, 2♂1♀), and FD-2 (N ​= ​3, 1♂2♀) through caudal intravenous injection. Syn1 was labeled by green fluorescent signal, α-BTX was labeled by red fluorescent signal, and the two signals were merged. Innervated NMJ displayed a yellow signal if green and red signals colocalized. Scale bar: 20 ​μm. **(b)** Quantification of NMJ integrity rate (in percentage) of colocalized signals. The percentage of Syn1/α-BTX colocalization of each sample was averaged from four different immunofluorescence-stained fields of view from the one individual sample. Data of each experimental group were averaged from three independent animal samples and presented as mean ​± ​SD (N ​= ​3). Total groups were analyzed and compared using MetaMorph software. One-way ANOVA was used for statistical analysis (∗∗∗, p ​< ​0.001).

To examine the integrity of NMJ, the axonal termini of MNs were detected by immunostaining. Antibody against Syn1 labeled with green fluorescent signal was to detect axonal terminal, while antibody against α-BTX labeled with red fluorescent signal was to detect the Acetylcholine receptor on motor endplates (Fig. 7a). We employed gastrocnemius muscle samples dissected from mice to visualize the spatial overlap of signals. Then, percentages of those presenting a colocalized yellow signal (green-labeled Syn1 overlapped with red fluorescent-labeled α-BTX) vs. those presenting only a red signal were calculated. The percentages of colocalized signals of Syn1 and α-BTX in Pgk1-, FD1-and FD2-injected SOD1-G93A mice were 79.3 ​± ​2.0 ​%, 70.7 ​± ​1.7 ​% and 71.5 ​± ​1.4 ​%, respectively (Fig. 7b). These values were significantly higher than the value of colocalized signals of Syn1 and α-BTX in PBS-injected mice (40.1 ​± ​10.4 ​%) (Fig. 7b). These results suggest that SOD1-G93A ALS-mice injected with FD-1 or FD-2 could preserve NMJ integrity longer than that of control mice, thus delaying NMJ denervation.

**Fig. 8:**
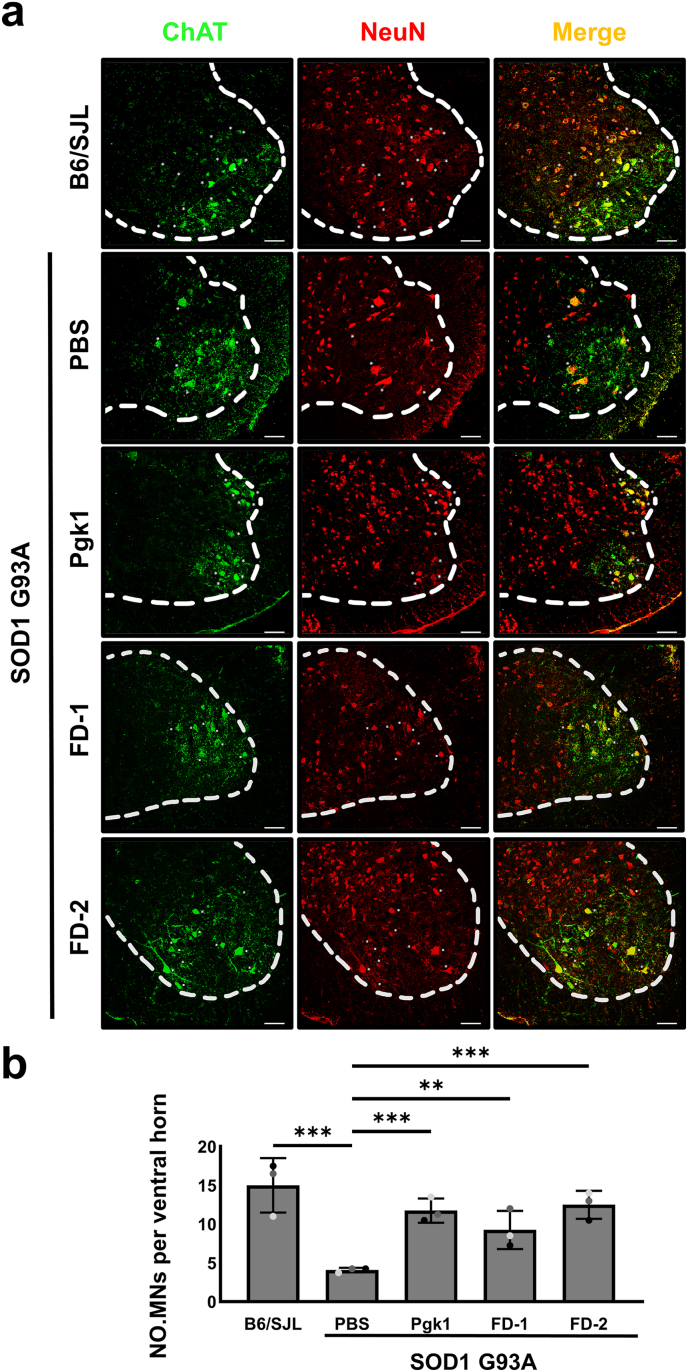
**The number of surviving MNscell bodies in the ventral horn of spinal cord was preserved in ALS-mice injected with either Pgk1 or each short FD peptide.(a)** Images were observed under confocal microscopy after dissection of L5 spinal nerves of 100 pnd old B6/SJL (strain control) (N ​= ​3, 1♂2♀) and ALS-mice, followed by immunohistochemical staining. PBS (N ​= ​3, 2♂1♀), Pgk1 (N ​= ​3, 1♂2♀), FD-1 (N ​= ​3, 1♂2♀) and FD-2 (N ​= ​3, 1♂2♀) were individually administered by intravenous injection into 60 pnd SOD1-G93A ALS-mice through the tail vein. ChAT was labeled by green fluorescent signal, and NeuN was labeled by red fluorescent signal with the merge of both signals indicating cell bodies of MN. Scale bar: 100 ​μm. **(b)** Cell bodies of MN (white asterisks). For each sample, the number of MNs colabeled with ChAT and NeuN was calculated as the average from four randomly selected fields of view. Quantification of the number of surviving MN cell bodies was averaged from three independent samples and presented as mean ​± ​SD (N ​= ​3). Statistical analysis was performed using a mixed-effects model (repeated-measures ANOVA) (∗∗, p ​< ​0.005; ∗∗∗, p ​< ​0.001).

Next, we counted the number of MNs cell bodies, or soma, that could generate electrical signals to control ventral horn. To accomplish this, we performed immunostaining to detect ChAT expressed in the cytosol of MNs labeled with green fluorescent signal (Fig. 8a). To detect NeuN, nuclei were labeled with red fluorescent signal (Fig. 8a). MN cell bodies were found to exhibit a yellow signal (green overlapped with red signals) (Fig. 8a). In B6/SJL mice (littermate control), cell bodies of ventral horn MNs in the L5 segment of spinal cord numbered 15.0 ​± ​3.5. The MN cell bodies in SOD1-G93A ALS-mice decreased in number to 4.1 ​± ​0.3 (Fig. 8a and b). However, MN cell bodies in SOD1-G93A ALS-mice injected with Pgk1, FD-1 and FD-2 numbered 11.8 ​± ​1.6, 9.3 ​± ​2.4, and 12.5 ​± ​1.8, respectively (Fig. 8a and b). Therefore, similar to Pgk1 injection, intravenous injection of each short FD peptide also effectively preserved a higher number of surviving MN cell bodies in the spinal cord of SOD1-G93A ALS-mice.

### ALS-mice injected intravenously with each short FD peptide exhibited stronger grip, increased mobility, and prolonged survival

**Fig. 9:**
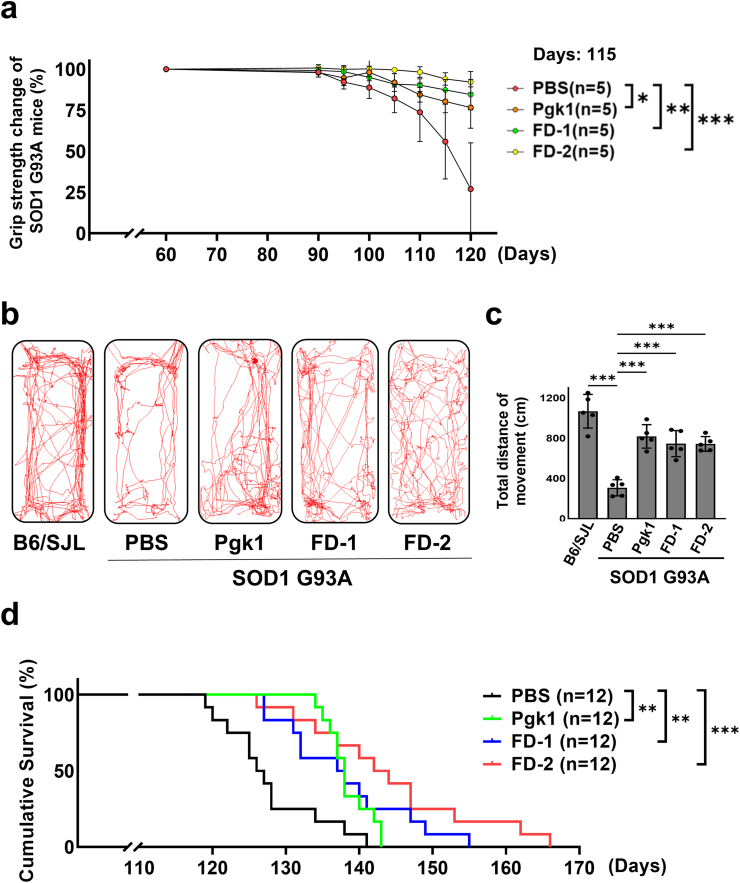
**Delay of symptom onset, increase of mobility and prolongation of survival of ALS-mice injected intravenously with each short FD peptide.(a)** Dynamic change of grip strength of SOD1-G93A ALS-mice aged between 60 and 120 pnd from four groups: PBS (N ​= ​5, 3♂2♀), Pgk1 (N ​= ​5, 2♂3♀), FD-1 (N ​= ​5, 3♂2♀) and FD-2 (N ​= ​5, 2♂3♀). Grip strength was averaged from total 60-pnd-old examined mice set as 100 ​%. At 115 pnd, grip strength shown in the Pgk1-, FD-1-, and FD-2-injected groups exhibited statistically significant differences compared to that of the PBS-injected control group. **(b)** Moving trajectories were recorded from B6/SJL (littermate control) (N ​= ​5, 3♂2♀) and SOD1-G93A ALS-mice injected with PBS (N ​= ​5, 2♂3♀), Pgk1 (N ​= ​5, 2♂3♀), FD-1 (N ​= ​5, 3♂2♀) and FD-2 (N ​= ​5, 3♂2♀), as indicated, at 124 pnd old. **(c)** Moving distance (in cm) of each group, as indicated, was quantified (N ​= ​5). **(a, c)** The value of each group was averaged from five independent samples and presented as mean ​± ​SD. One-way ANOVA was used for statistical analysis (∗, p ​< ​0.01; ∗∗, p ​< ​0.005; ∗∗∗, p ​< ​0.001). **(d)** Cumulative survival of SOD1-G93A ALS-mice treated with PBS (N ​= ​12, 7♂5♀), Pgk1 (N ​= ​12, 6♂6♀), FD-1 (N ​= ​12, 6♂6♀) and FD-2 (N ​= ​12, 5♂7♀) was calculated. ALS-mice were randomly dispatched to different treatment groups without sex bias. The log-rank (Mantel-Cox) test was used to determine survival curves (Kaplan-Meier curves).

We asked if peptide FD-1- or FD-2-injected SOD1-G93A ALS-mice could delay the onset of ALS symptoms. First, we examined the grip strength of ALS-mice aged 60 to 125 pnd. When the grip strength of 60-pnd-old ALS-mice was set as 100 ​%, we found that it gradually decreased down to 55.8 ​± ​22.8 ​% in 115-pnd-old PBS-injected ALS-mice (Fig. 9a). Nevertheless, the median grip strength values of 115-pnd-old and beyond ALS-mice injected with Pgk1, FD-1 and FD-2 were 80.2 ​± ​6.9 ​%, 87.4 ​± ​6.4 ​% and 94.1 ​± ​3.8 ​%, respectively (Fig. 9a).

Next, we monitored the moving trajectories in 124-pnd-old ALS-mice (Fig. 9b). We found that the moving distance for PBS-injected ALS-mice was significantly reduced compared to that of B6/SJL (littermate control), e.g., 307.1 ​± ​79.9 ​cm vs. 1067.0 ​± ​166.5 ​cm (Fig. 9c). However, the moving distance of Pgk1-, FD-1- and FD-2-injected 124-pnd-old ALS-mice exhibited significantly longer moving distance than that of PBS-injected ALS-mice, e.g., 817.8 ​± ​116.3 ​cm, 745.8 ​± ​129.5 ​cm and 743.4 ​± ​73.2 ​cm, respectively (Fig. 9c). Moreover, we demonstrated that the survival days of PBS control, Pgk1-, FD-1- and FD-2-injected groups ranged from 119 to 141 pnd, 134 to 143 pnd, 128 to 155 pnd and 127 to 167 pnd, respectively (Fig. 9d). The overall Kaplan-Meier survival curves displayed a consistent rightward shift and presented statistical significance with the log-rank test. These findings suggest that the cumulative survival of Pgk1-, FD-1- and FD-2-injected SOD1-G93A mice was higher when compared to the survival days of PBS-injected ALS-mice (Fig. 9d).

Overall, intravenous injection of full-length 417-aa Pgk1 and each Pgk1-derived 16-aa short FD peptide elicited similar results by maintaining grip strength, improving mobility, and prolonging lifespan of ALS-mice compared to the control group.

## Discussion

### Interaction between an FD-containing key segment of the C-terminal 325–417 aa region of ligand ePgk1 and the C-terminal 404–431 aa region of receptor Eno2 improves NOMNs by triggering a specific pathway

Previously, we reported that Eno2 functions as a receptor on the neuronal membrane to receive signals from the secreted ligand ePgk1 [16]. This signaling attenuates the p-P38/p-Limk1-S323 axis, as detailed in the Introduction, thereby reducing the expression of p-Cofilin [16]. The C-terminal segment (residues 404–433) of Eno2 has been identified as critical for promoting neuronal survival, activating neurite outgrowth and stabilizing the microtubule network, as well as facilitating the translocation of Eno2 to the cell membrane [27,28]. Moreover, through cell surface crosslinking and immunoprecipitation, we confirmed that the C-terminal segment 325–417 of ligand ePgk1 interacts with the C-terminal region 404–431 of receptor Eno2 [16].

In this study, we performed domain mapping of full-length Pgk1 through serial deletions. Results showed that the key functional domain of ePgk1 resides at C-terminal segment 345–360. Indeed, this segment is retained within both short peptides, accounting for their ability to boost NOMNs in a manner equivalent to that of ePgk1. Meanwhile, Lee et al. demonstrated that the 419th aspartic acid of receptor Eno2 is a key aa affecting NOMNs mediated by ePgk1-Eno2 interaction [29]. Docking analysis predicted that the 353rd lysine (K353) of Pgk1 could be a major corresponding residue for D419. Both short FD peptides also contain K353 of Pgk1. Therefore, we hypothesized that promoting electrostatic interaction between the positively charged K353 lysine of Pgk1 and negatively charged D419 aspartic acid of Eno2 might be a critical factor contributing to the mediation of NOMNs in NSC34 ​cells and MNs function in ALS-like animals.

Notably, this novel neuroprotective domain within the Pgk1 is independent of any conventional domains harboring a previously identified intracellular function, such as substrate binding domain (segment 1–145), ADP-binding domain (segment 146–417) and catalytic site D375 involved in Pgk1 glycolysis [30]. All of above these known domains can be excluded from the minimally functional neuroprotective domain discovered in this study. This finding further supports the non-canonical role of ePgk1 in mediating NOMNs, as reported by Lin et al. [15].

### Reduced p-Cofilin expression favors actin dynamics, resulting in improving symptoms of neurodegenerative diseases

NOMNs derived from NSC34 neural cells was used in this study to evaluate the neuroprotective ability of ePgk1 and short peptides *in vitro*. Here, we recapitulate the activity of Cofilin and phospho-Cofilin in the context of axonal inhibition and growth. First, phosphorylation of Cofilin inactivates it, leading to the stabilization and accumulation of F-actin. This causes a decrease in actin dynamics and stalled NOMNs. Nevertheless, Cofilin is directly involved in the pathogenesis of several neurodegenerative diseases [31]. For example, a factor contributing to the pathological state is C9orf72, a common genetic cause of ALS. That is, the reduced function of C9orf72 increases p-Cofilin which leads to neurological dysfunction. The loss function of C9orf72, which normally interacts with Cofilin to regulate axonal actin dynamics, impairs axonal growth, actin dynamics, and cargo trafficking along the actin cytoskeleton at synapses [32,33]. It is also associated with impaired neural growth cone motility and guidance, synaptic dysfunction, dendritic spine loss, and neuronal plasticity deficits [31,32]. Indeed, upregulated p-Cofilin has been detected in lymphoblastoid cells, postmortem brain samples and C9ORF72-depleted MNs of patients with ALS. This suggests that C9ORF72 regulates small GTPases, affecting actin dynamics in axons [33]. Furthermore, significantly increased levels of F-actin have been detected in spinal cord neurons from sporadic patients with ALS and in TDP-43 rNLS mouse neurons. To explain, a change in the normal aa sequence of TDP-43 occurs by mutated nuclear localization signal (rNLS) whereby the protein cannot be transported to the cell nucleus. Such mislocalization can lead to disease states by the accumulation of cytoplasmic TDP-43 in certain neurodegenerative disorders like ALS where proper nuclear localization is essential [34]. As explained above, increased phosphorylation of Limk1 inactivates Cofilin. Interestingly, in the same samples, increased phosphorylation of Limk1 and Cofilin was also observed, indicating that p-Cofilin is strongly associated with ALS/TDP-43 pathology. It is worth noting that Jasplakinolide-induced actin polymerization was found to promote the mislocalization of TDP-43 to the cytoplasm. This finding supports the linkage between ALS and TDP-43. It also confirms that abnormal nucleocytoplasmic shuttling of TDP-43 is associated with disrupted actin dynamics in ALS.

Second, active, or dephosphorylated Cofilin, allows actin turnover which promotes NOMNs. In the present study, we found that the short FD peptides recapitulate the signaling pathway whereby ePgk1 interacts with Eno2 to suppress p-P38/p-MK2/p-Limk1-S323 without altering the phosphorylation level of p-Limk1-T508. This activity results in decreasing p-Cofilin.

As previously noted, supplementary short FD peptide reverses the behavior of inactive Cofilin in its phosphorylated state. That is, reduced p-Cofilin favors actin dynamics, neural trafficking and structural integration. Thus, administration of short FD peptide could improve NOMNs, reduce TDP-43 cytoplasmic mislocalization, and alleviate p-Tau-S396 accumulation in ALS-like NSC34 ​cells. Moreover, administration of short FD peptide could increase the formation of branched CaPMNs in zebrafish embryos and induce higher rescue of axonal growth and motor function in ALS-like zebrafish larvae. Thus, it can be concluded that both full-length ePgk1 and short FD peptides act through the same regulatory pathway to halt the progression of ALS, as determined by pathophysiological studies of animal models.

### Neuroprotective effects of full-length ePgk1 and two short FD peptides in ALS-like MN cells (*in vitro*) and ALS animal models (*in vivo*)

Stevens et al. reported that p-Tau-S396 accumulates in MNs in ALS, contributing to mitochondrial fragmentation and oxidative stress [25]. Cytoplasmic aggregates of TDP-43 can disrupt actin dynamics and lead to ALS pathology [34,35]. Here, we demonstrated that addition of each short FD peptide can reverse the pathophysiological effects triggered by p-Tau-S396 accumulation and TDP-43 cytoplasmic mislocalization to improve survival of ALS-like NSC34 ​cells under oxidative stress.

To confirm these results *in vivo*, we employed transient transgenic ALS-like zebrafish. To better understand this model, we cite the work of Kalil and Dent who demonstrated that cell membrane protrusion during axonal branching requires accumulated actin filaments (F-actin) beneath the plasma membrane to form patches [36]. Membrane protrusion also requires actin nucleation, actin branching and actin elongation. When actin bundles extend outward forming filopodia, microtubules are cleaved by Spastin and Katanin to then enter the nascent branch which continues to mature and extend axonal branching. In this process, active Cofilin is important for the turnover of actin filaments. This increases the pool of free actin monomers, but it is not the driver of axonal growth. Instead, the axonal branching phenotype observed in zebrafish embryos results from cytoskeletal remodeling caused by additional F-actin formation. F-actin formation is permitted by the reduced level of p-Cofilin driven by the ePgk1-Eno2-elicited signaling discussed above. Indeed, we observed increased axonal branching of MNs in ePgk1/Eno2-overexpressing embryos [15]. This finding is further validation that ePgk1/Eno2 interaction can reduce p-Cofilin level and that such reduction causes an increase in the formation of actin filaments. The resultant cytoskeletal remodeling then promotes increased NOMNs. This means that ectopic axonal branching, such as that observed in zebrafish embryos administered with ePgk1 or short peptides, could be attributed to the reduced level of p-Cofilin in MNs by potentiating the formation of activated actin filaments. Thus, increased ectopic axonal branching, improved AG, and increased MNs function, as demonstrated in the ALS-like zebrafish embryos treated with short FD peptide, are the main physiological outcomes of the signaling axis we describe as p-Pak1-T423/p-P38-T180/p-MK2-T334/p-Limk1-S323/p-Cofilin-S3.

Additionally, we found that the structural integrity of NMJ in ALS-mice treated with each short FD peptide is substantially preserved, even at 75 pnd old. We also saw an increased number of MNs cell bodies in the ventral horn of spinal cord at 100 pnd old and improved locomotion at 124 pnd old. These findings confirmed that administration of FD can delay disease progression and prolong the lifespan of SOD1-G93A mice. This was demonstrated by the high degree of autonomous movement and feeding ability, even at 124 pnd old, the typical endpoint for SOD1-G93A ALS-mice. Results also indicated that FD is more effective in slowing the decline in muscle function compared to the result previously reported by Lin et al. who tested intra-gastrocnemius muscular injection of ePgk1 on ALS-mice [15,16]. Importantly, we demonstrated equivalent efficacy between both short peptides and full-length ePgk1.

### *In vitro* and *in vivo* comparison of neurologic improvement by short peptides FD-1 and FD-2

FD-1 and FD-2 share similar molecular weights, isoelectric points and identical aa compositions, except for one residue. However, we found that neurologic outcomes exhibited little different efficacy between them after *in vitro* or *in vivo* treatment. Specifically, FD-1 treatment performs better than FD-2 treatment *in vitro*, such as rescue of neurite length of NOMNs derived from NSC34 ​cells (Fig. 2a and b). On the other hand, FD-2 treatment performs better than FD-1 *in vivo*, such as promoting CaPMNs branching and suppressing p-Cofilin level in zebrafish (Fig. 6f and h), delaying soma apoptosis in the ventral horn of spinal cord (Fig. 8b), and prolonging survival in ALS mice (Fig. 9a and d). To explain, FD-1 contains alanine at the 10th position with a simple methyl side chain having minimal structural impact. In contrast, FD-2 contains proline at the 10th position with a pyrrolidine ring having restricted peptide flexibility. We speculate that proline can naturally undergo cis–trans isomerization, resulting in influencing protein folding and conformation [37]. It has been demonstrated that cis–trans isomerization of proline plays critical roles in cellular signal transduction, active membrane transport, enzymatic activity, and protein–protein interactions [[38], [39], [40]]. Moreover, incorporating proline into peptides can serve as an effective cosolvent [41], enhancing molecular stability and solubility, thereby improving distribution within plasma and interstitial fluids. Nonetheless, further analyses, such as enzyme degradation assays, pharmacokinetic studies, and structural modeling with docking simulations, are necessary to elucidate the mechanisms underlying differences between the *in vitro* and *in vivo* performance of FD-1 and FD-2.

### Therapeutic applications of full-length ePgk1 and its derived short FD peptides

Intracellular Pgk1 functions canonically as a glycolytic enzyme. It is, therefore, considered a bioenergetic protein that can bind substrates and products, such as MgADP or MgATP, thereby modulating motor neurons that lack glucose. Chaytow et al. [42] reported that either intracellular overexpression of Pgk1 or treatment with Terazosin (TZ) could increase the enzymatic activity of intracellular Pgk1 and, hence, ATP production. They found that this activity prevents oxidative stress-induced cell death, rescues stress granule assembly, and promotes neuronal repair to delay neuronal death. Thus, TZ treatment can improve MNs function and increase MNs numbers in ALS model zebrafish and mice, ultimately extending survival. Meanwhile, however, we demonstrated that extracellular Pgk1 functions non-canonically as a cross-tissue mediator between muscle and neurons [15]. We have previously explained that Pgk1-Eno2 interaction suppresses the p-P38/p-Limk1-S323 axis and reduces p-Cofilin [16]. Correspondingly, with identical functional domains, FD-1 and FD-2 can also reduce p-Cofilin by the same suppressive activity, thereby improving NOMNs of NSC34 neural cells. The same holds true for increased AG of ALS-like zebrafish and improved locomotive activity of ALS-mice, both achieved in a glycolysis-independent manner. Collectively, therefore, we conclude that either intra- or extracellular Pgk1 in either its canonical glycolysis-dependent or non-canonical glycolysis-independent roles, respectively, can provide therapeutic benefit in neurodegenerative diseases such as ALS.

Nevertheless, short peptides offer several advantages over full-length proteins, including more straightforward synthesis and purification, higher affinity, specificity, efficacy and bioavailability. They also exhibit versatile conformational structures with relatively low toxicity and fewer side effects. Lower manufacturing costs can be achieved through shorter production time and fewer cellular resources [43,44]. Moreover, ideal secondary structures can be constructed through chemical modification, resulting in enhanced bioactivity, including improved selectivity, stability, and solubility [45,46]. The two 16-aa short FD peptides reported in this study offer significant advantages for future research. These short peptides are also distinguished by their high translational potential within the context of innovations in AI-driven aa modification and delivery. Particularly, the therapeutic applicability and potential of these peptides hold promise for the treatment of ALS disease and lead to more substantial translational value in the neuroscience field.

## Author contributions

Conceptualization, CYL, BCL and HJT; data curation, CYL, BCL, PHZ, SCL and WZC; formal analysis, CYL, BCL and PHZ; funding acquisition, CYL and HJT; investigation, BCL, PHZ and SCL; methodology, CYL, BCL, PHZ and CCW; project administration, CYL and HJT; resources, CCW and HJT; software, SCL and BCL; supervision, HJT; visualization, CYL, SCL and BCL; writing-original draft, CYL, BCL and HJT; writing-review & editing, CYL and HJT.

## Funding sources

This study was supported by the 10.13039/100020595NSTC, Taiwan, under 111-2313-B-030-001, 112-2313-B-030-001, 112-2622-B-030-001 and 113-2313-B-030-001 to HJT and 112-2314-B-715-004-MY2 to CYL. This study was also supported by 10.13039/501100013784FJCU under 110-20010101, 111-20010101, 9991F02-1120232, 48010101112, 48010102112, 48010101113, 48010102113, and USA 10.13039/501100013784FJCU Alumni Foundation F630410 to HJT, as well as MacKay Medical University, MMC-RD-112-1B-P006 to CYL.

## Data sharing statement

All data are available in the main text or in the supplementary materials. There are no restrictions on material or data. Raw data are available through request to the corresponding author.

## Declaration of competing interest

The authors declare no conflict of interest.
