# Supplementary figures and images for: A 16-amino acid peptide delays the progression of motor neuron degeneration and pathogenic symptoms in ALS models
Source: Neurotherapeutics. 2025 Nov 26;23(1):e00806. doi: 10.1016/j.neurot.2025.e00806 (PMC12976506; doi:10.1016/j.neurot.2025.e00806)

Figure 1b

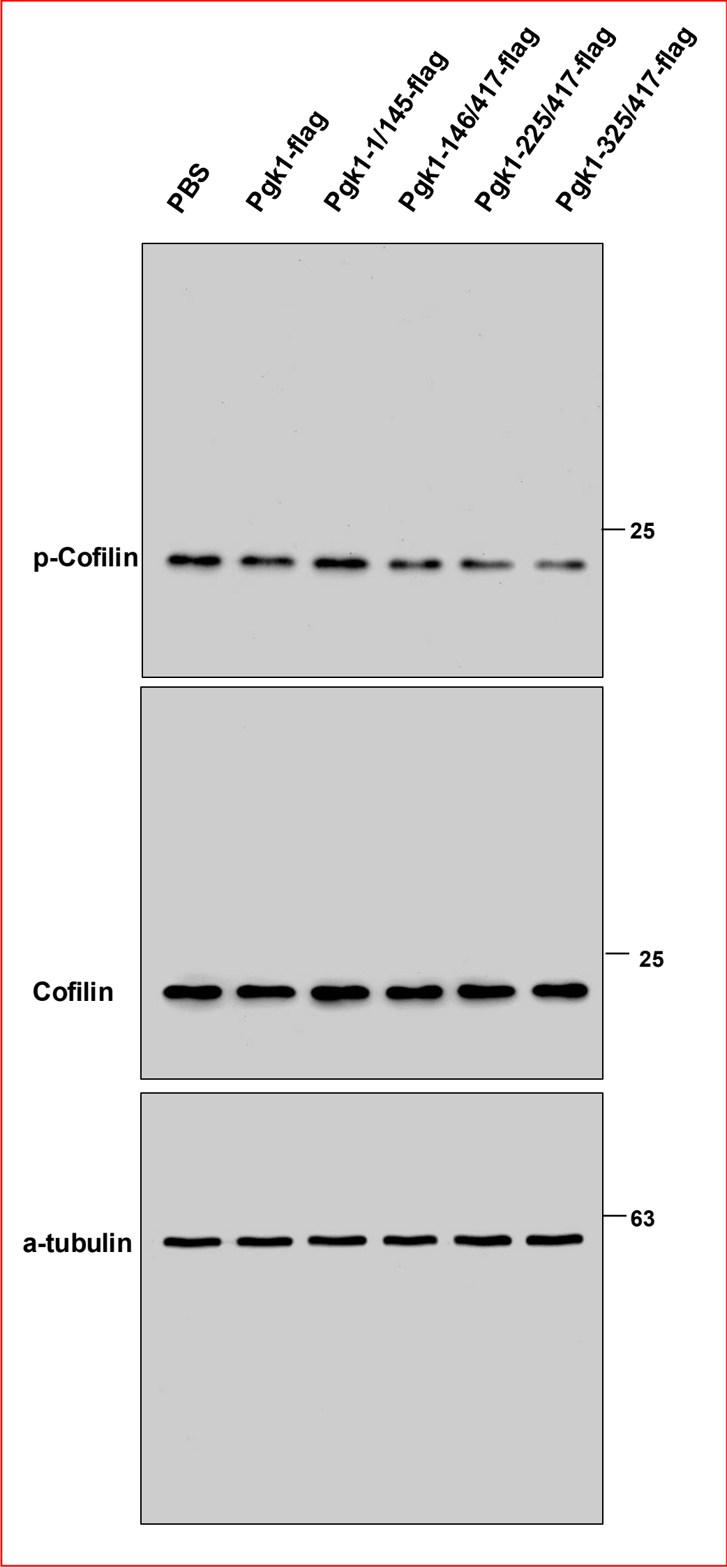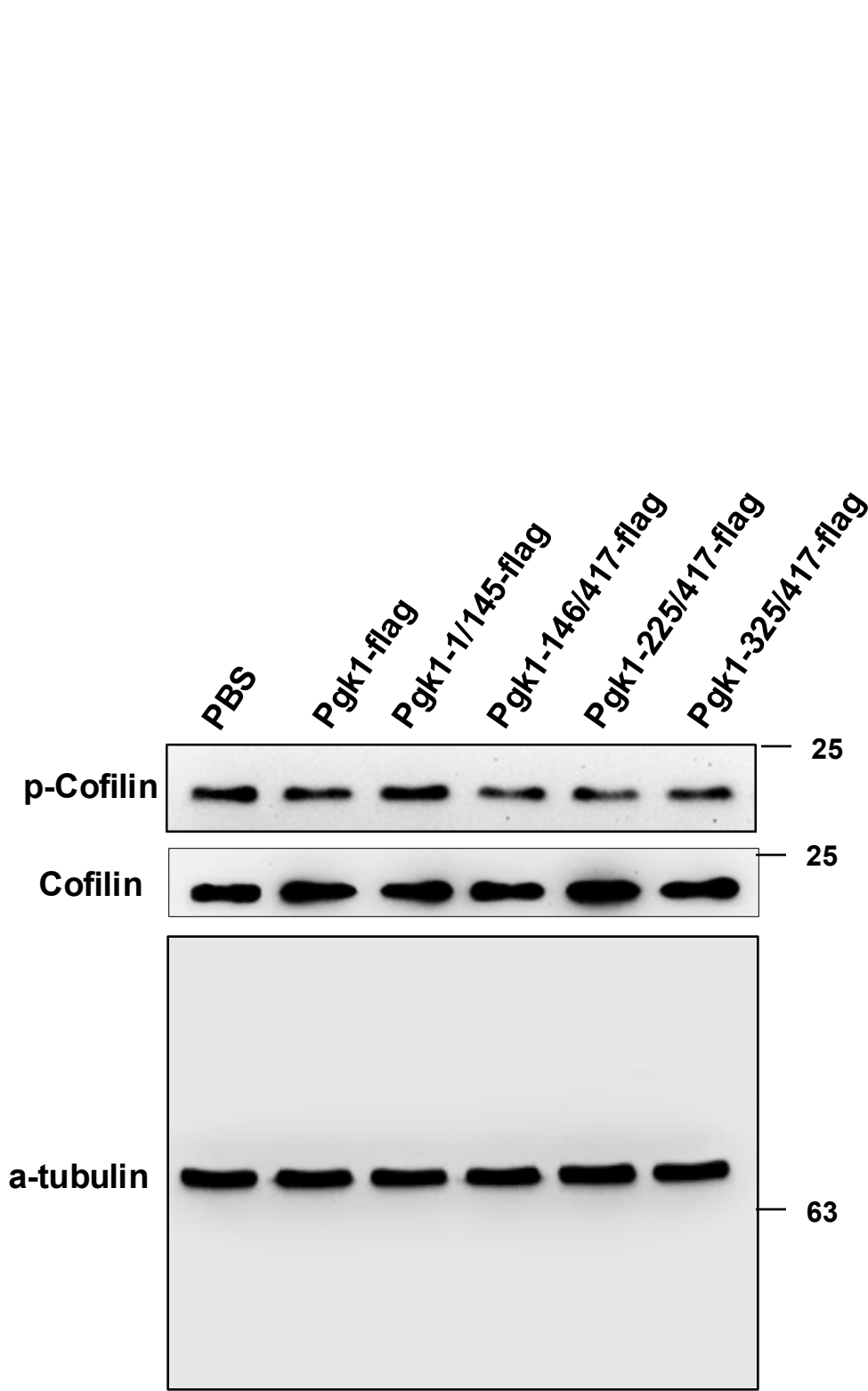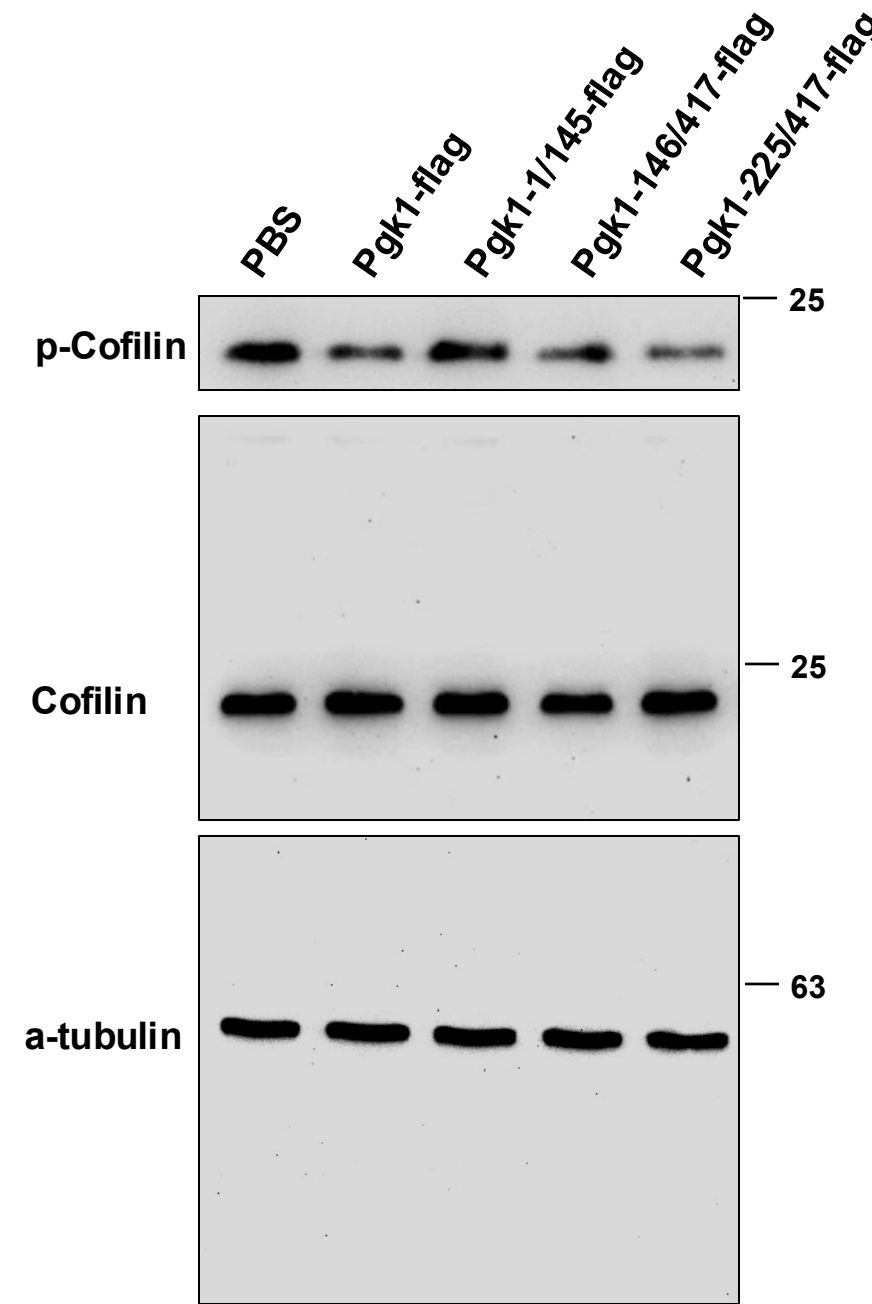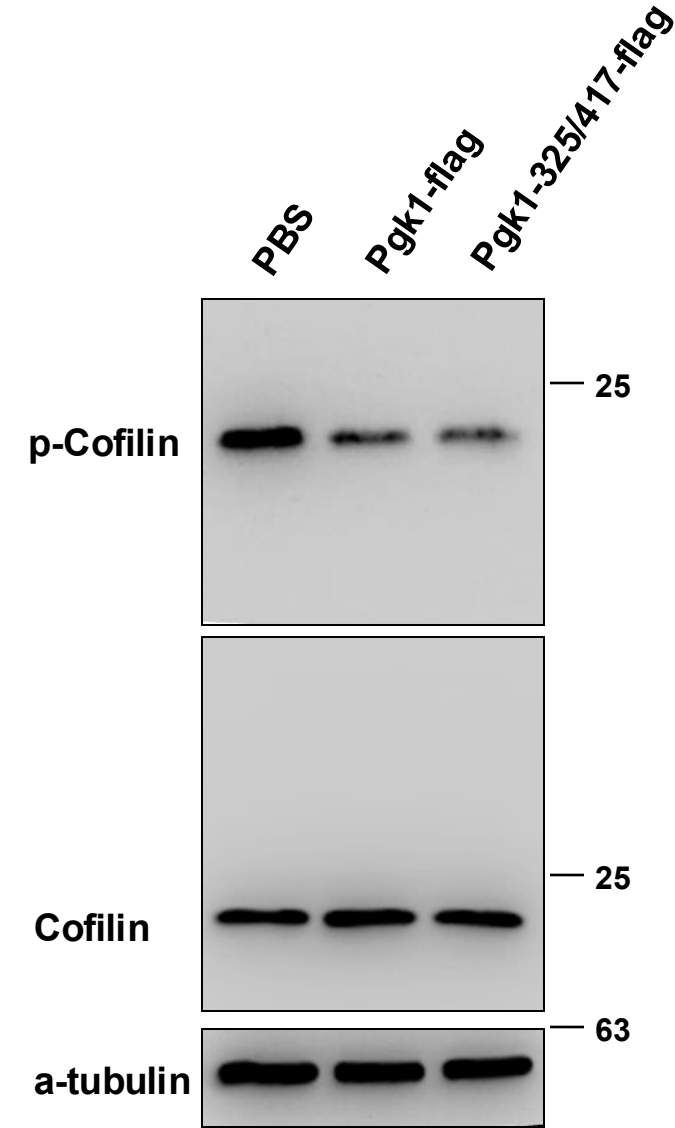

Figure 1c

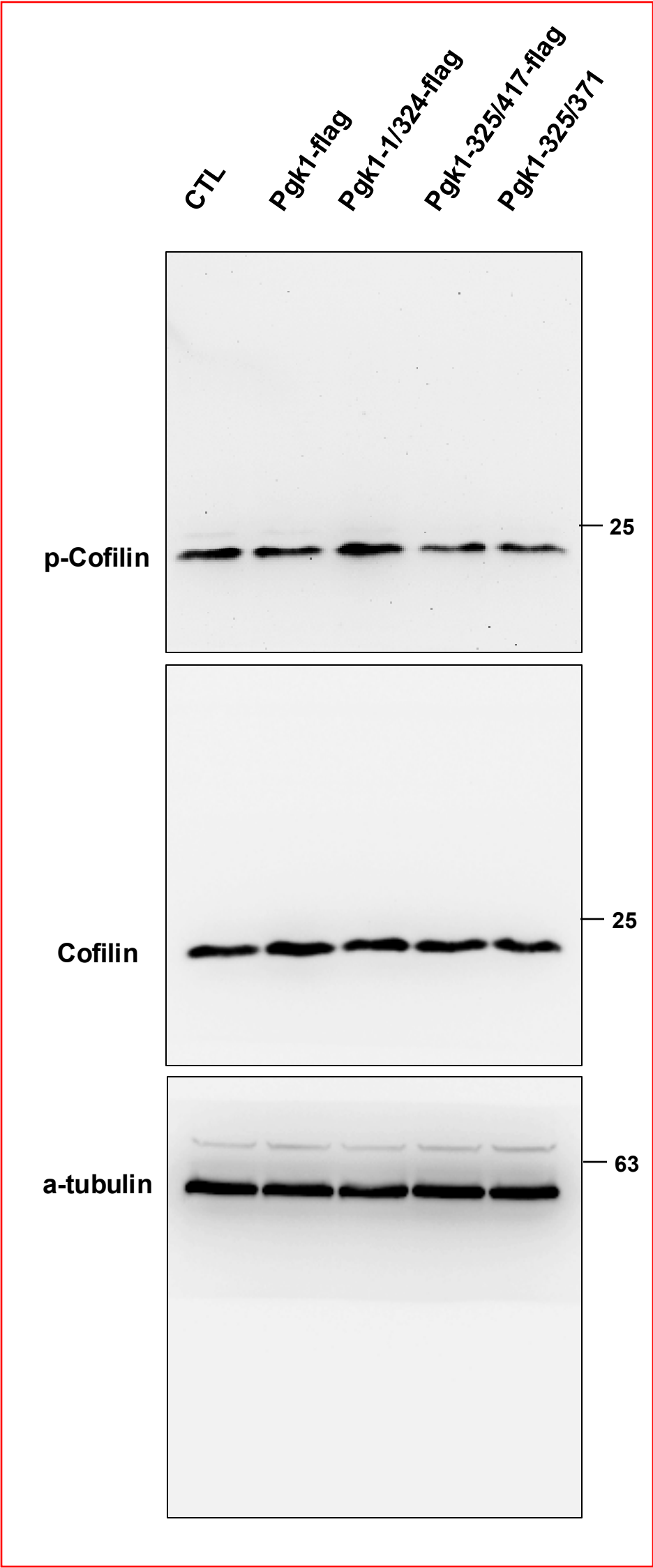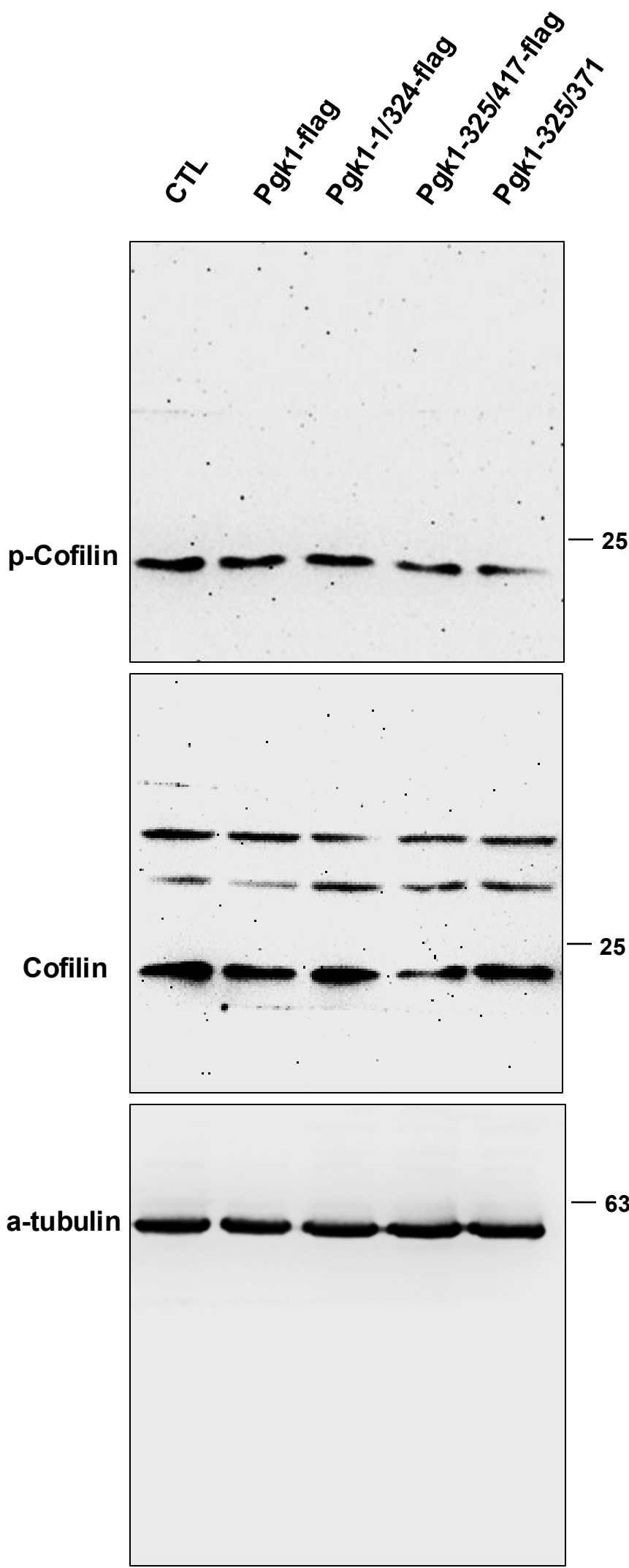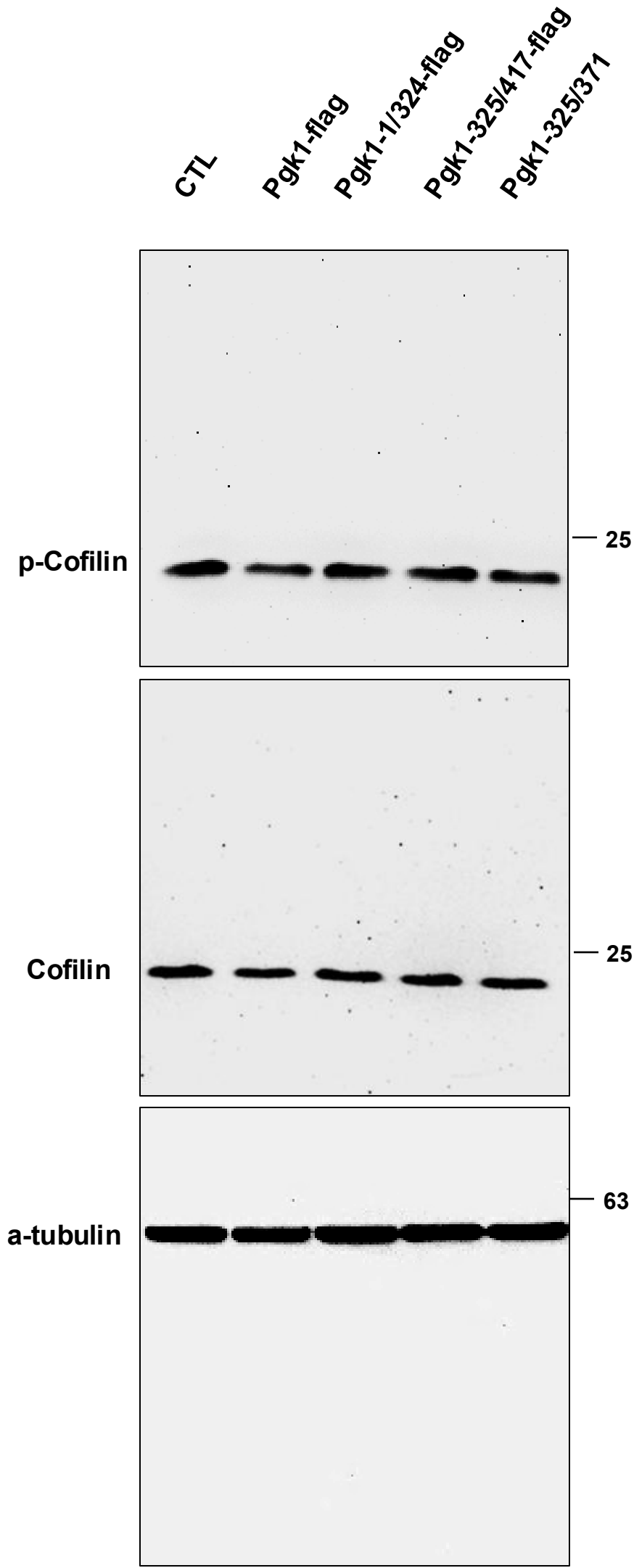

Figure 1d

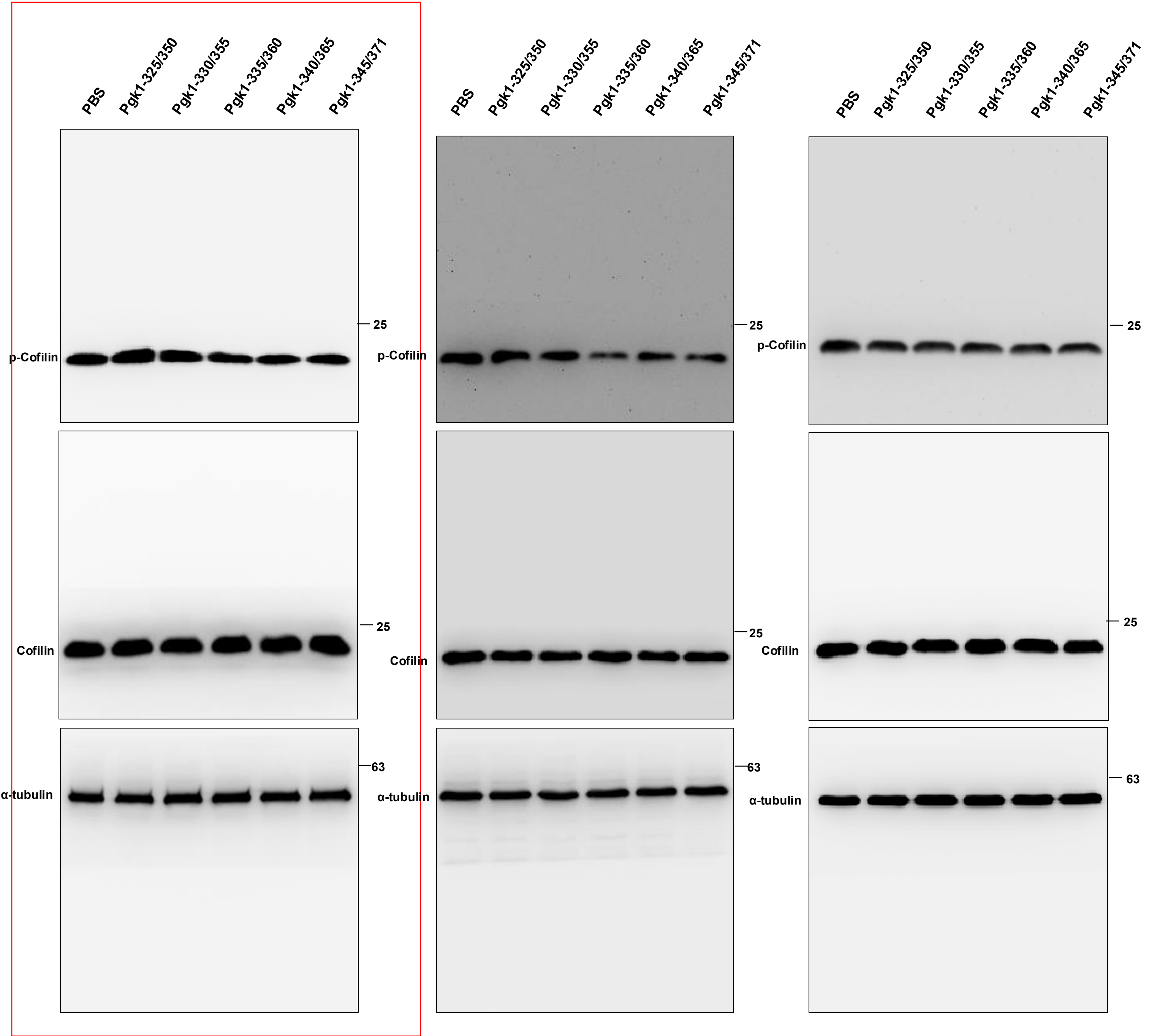

Figure 2c

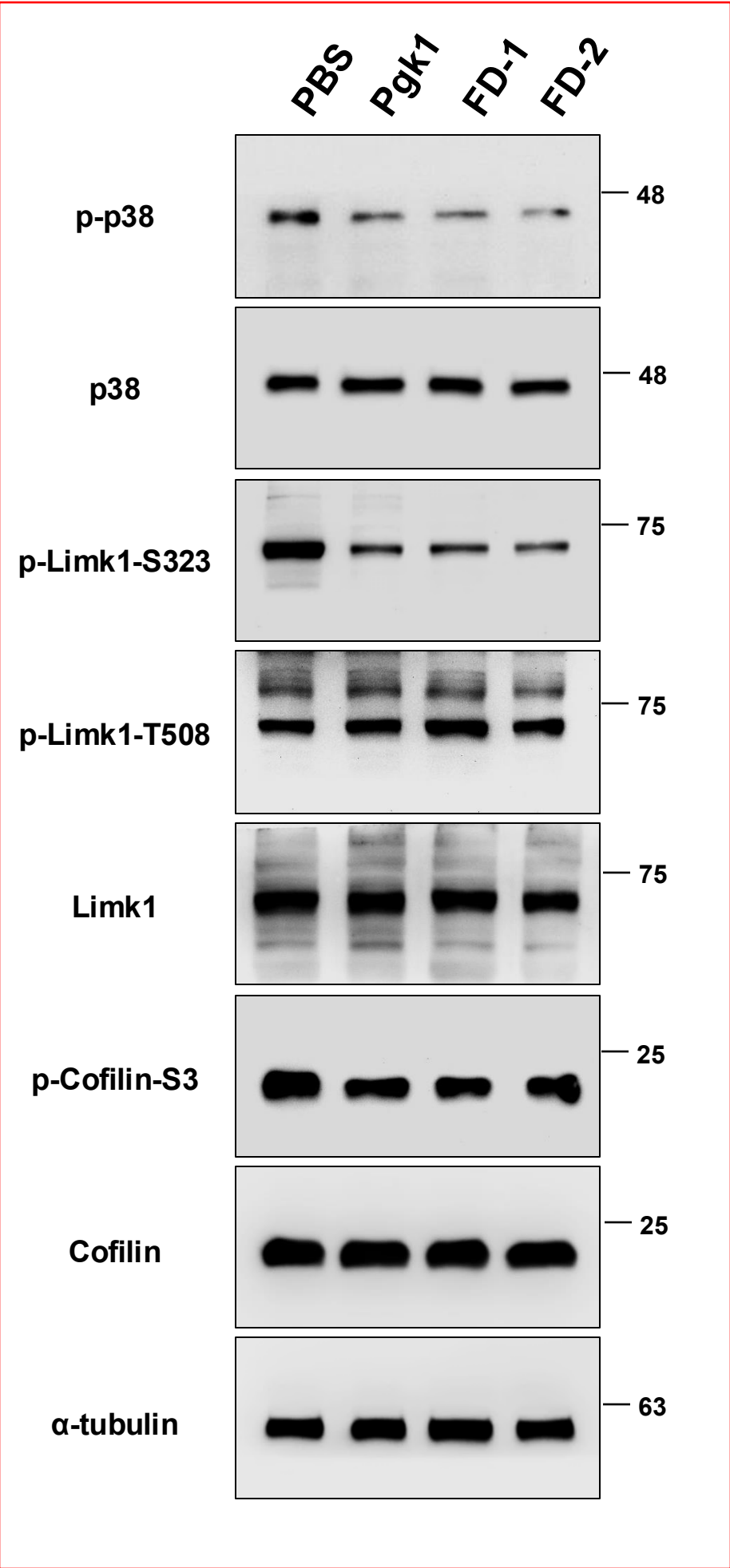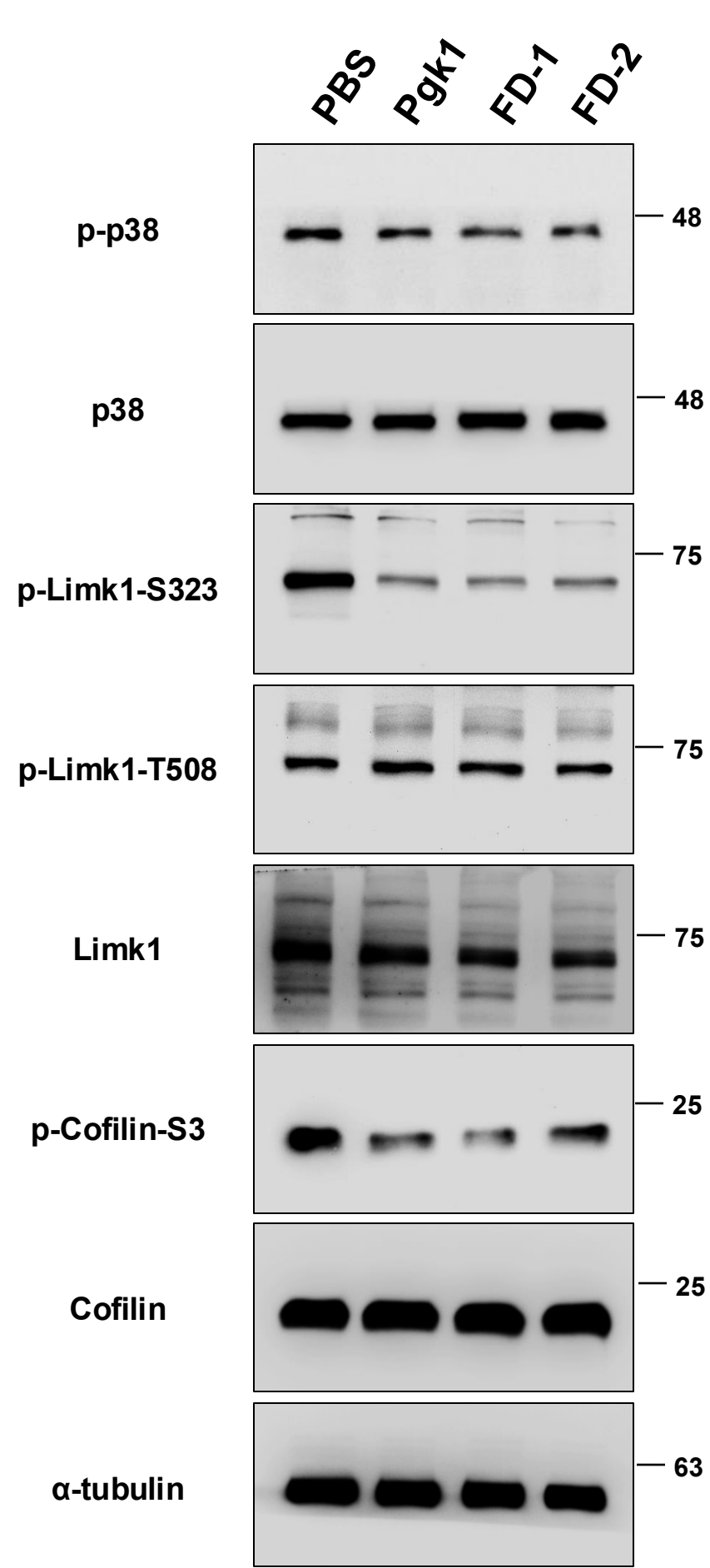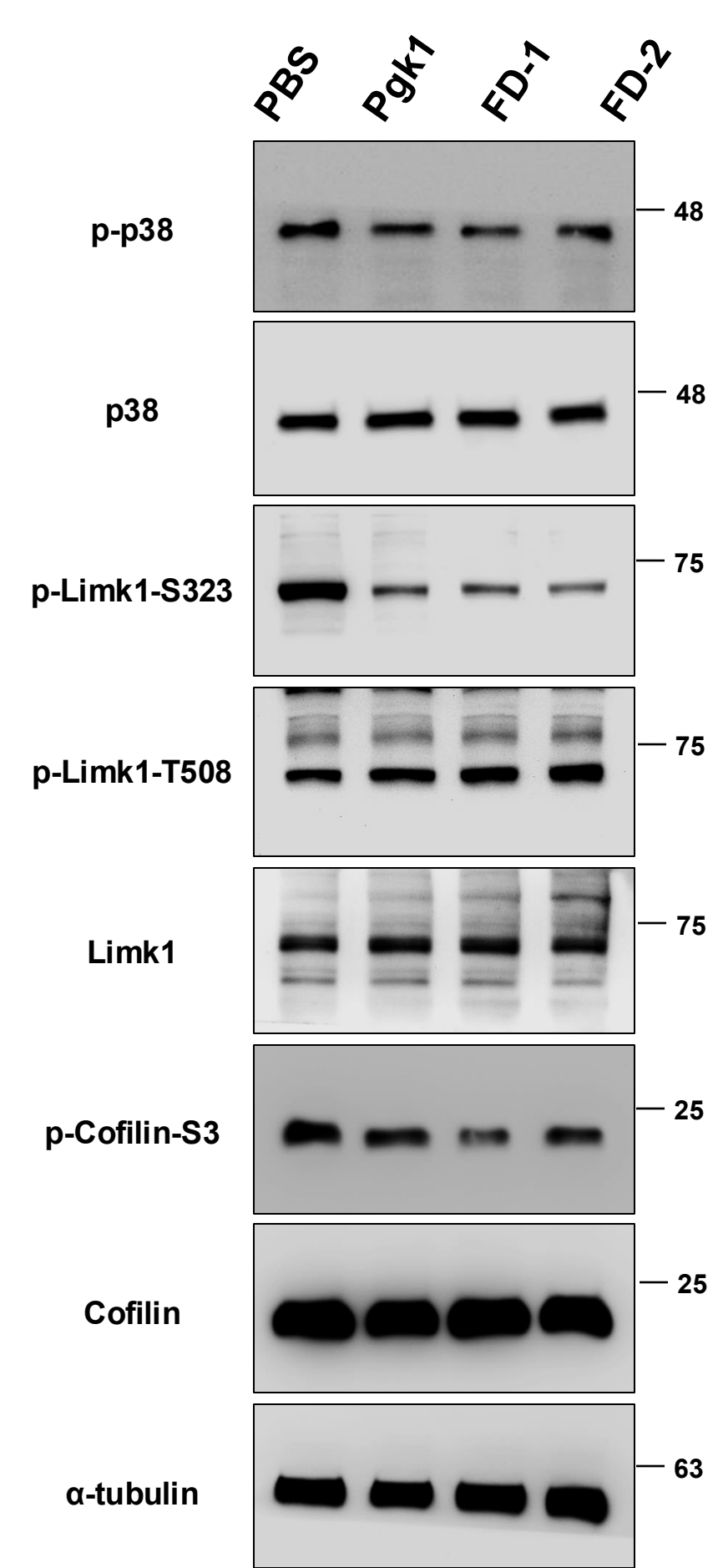

Figure 3a

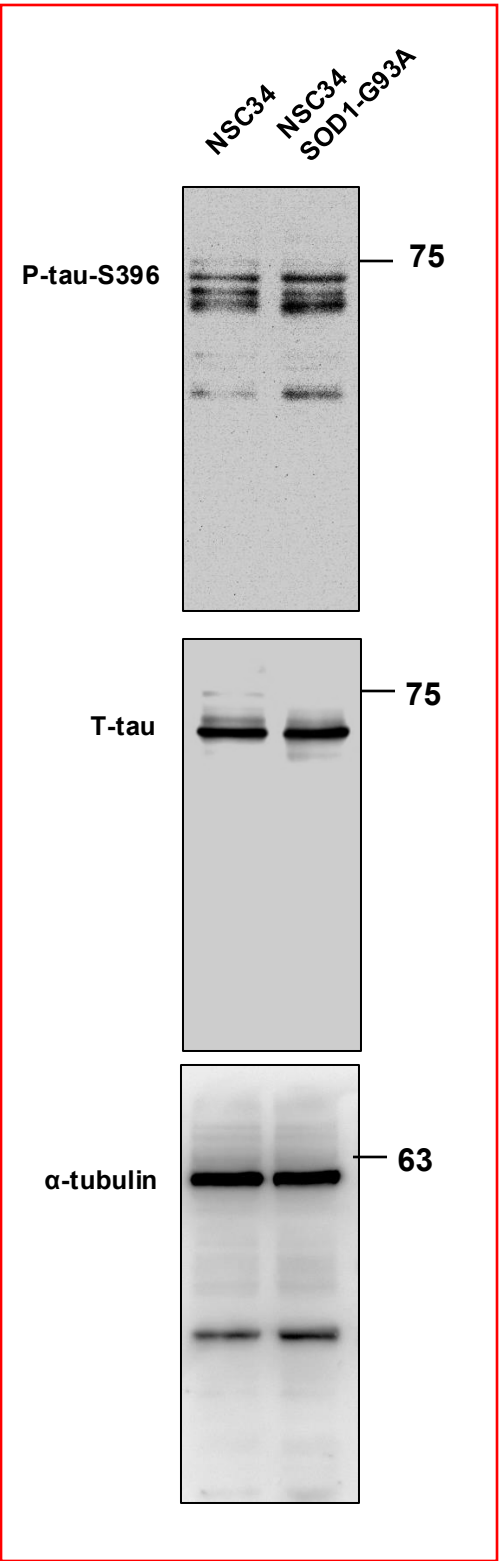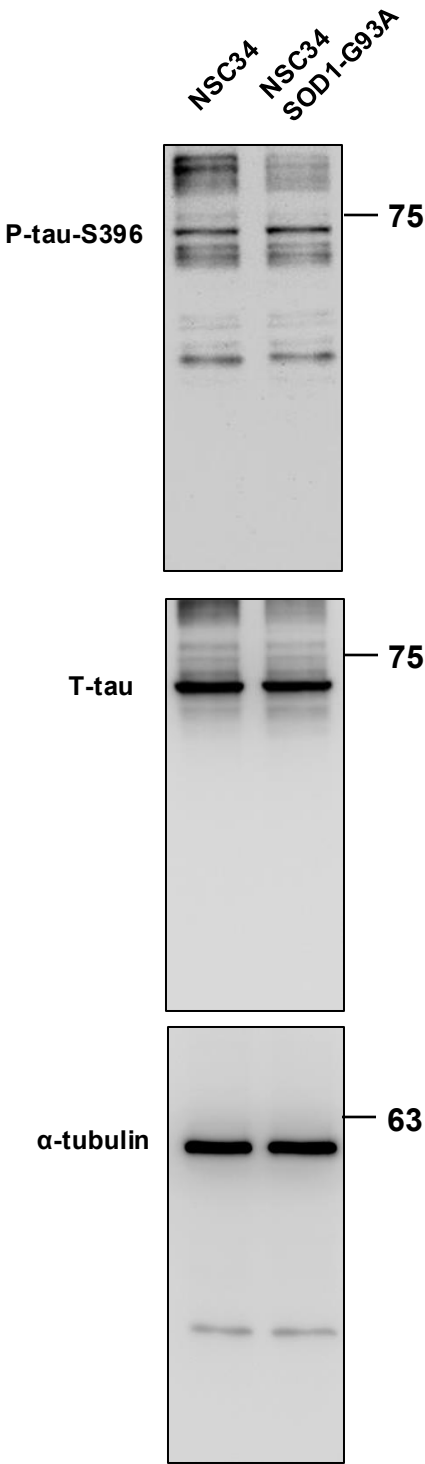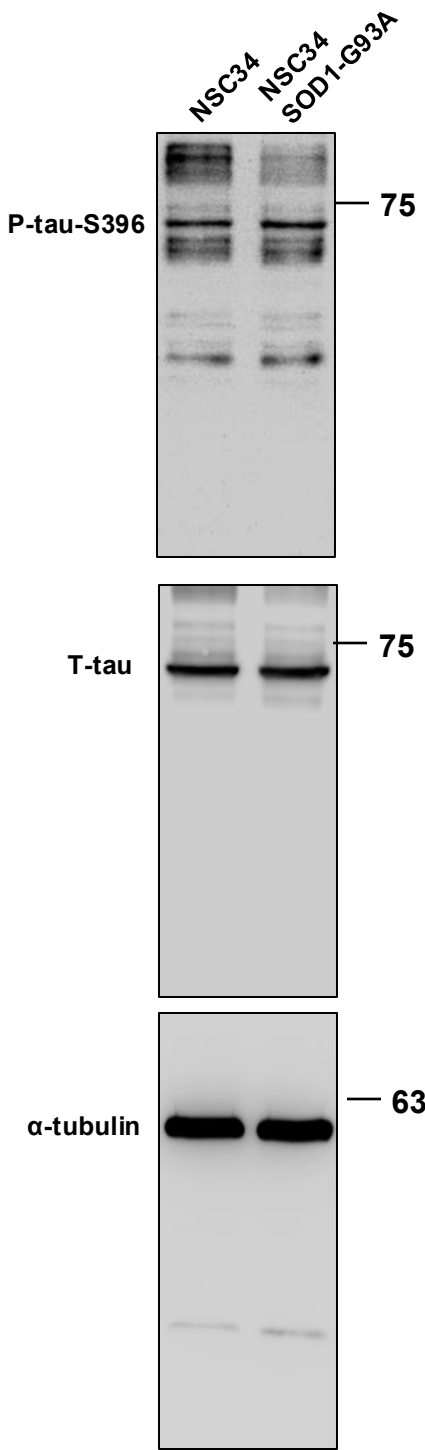

Figure 3c

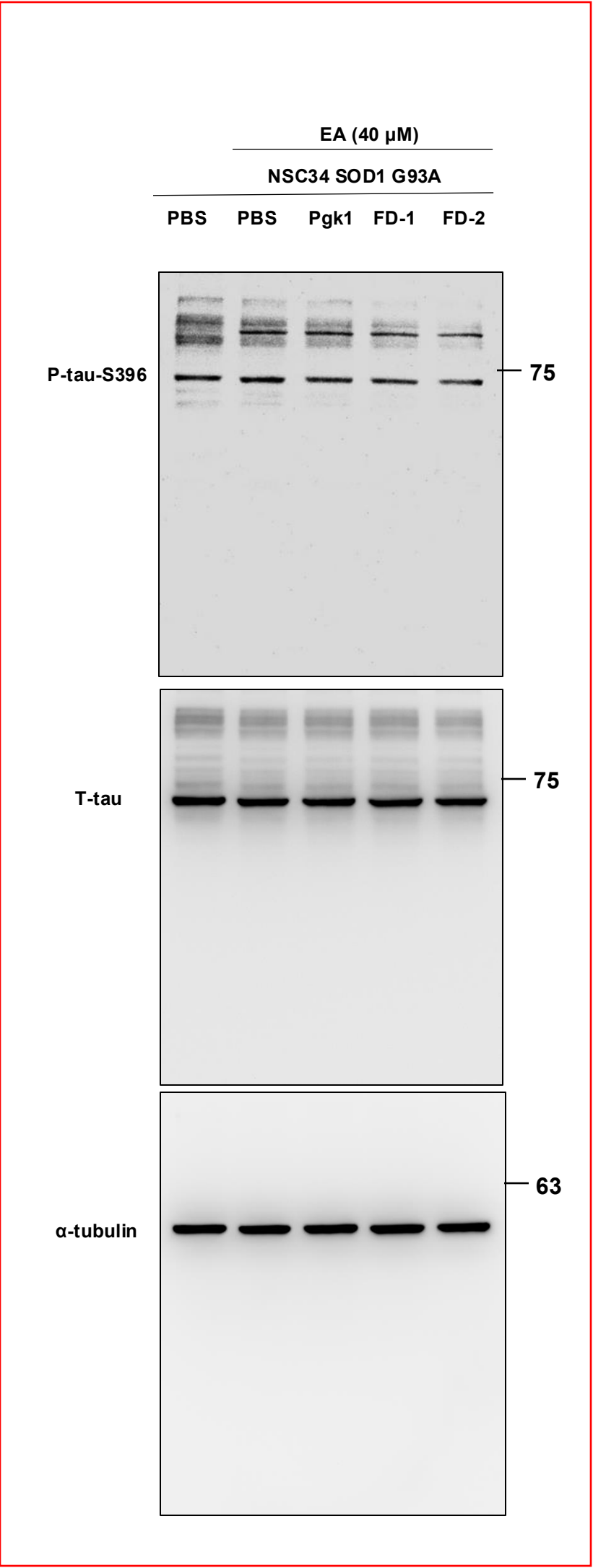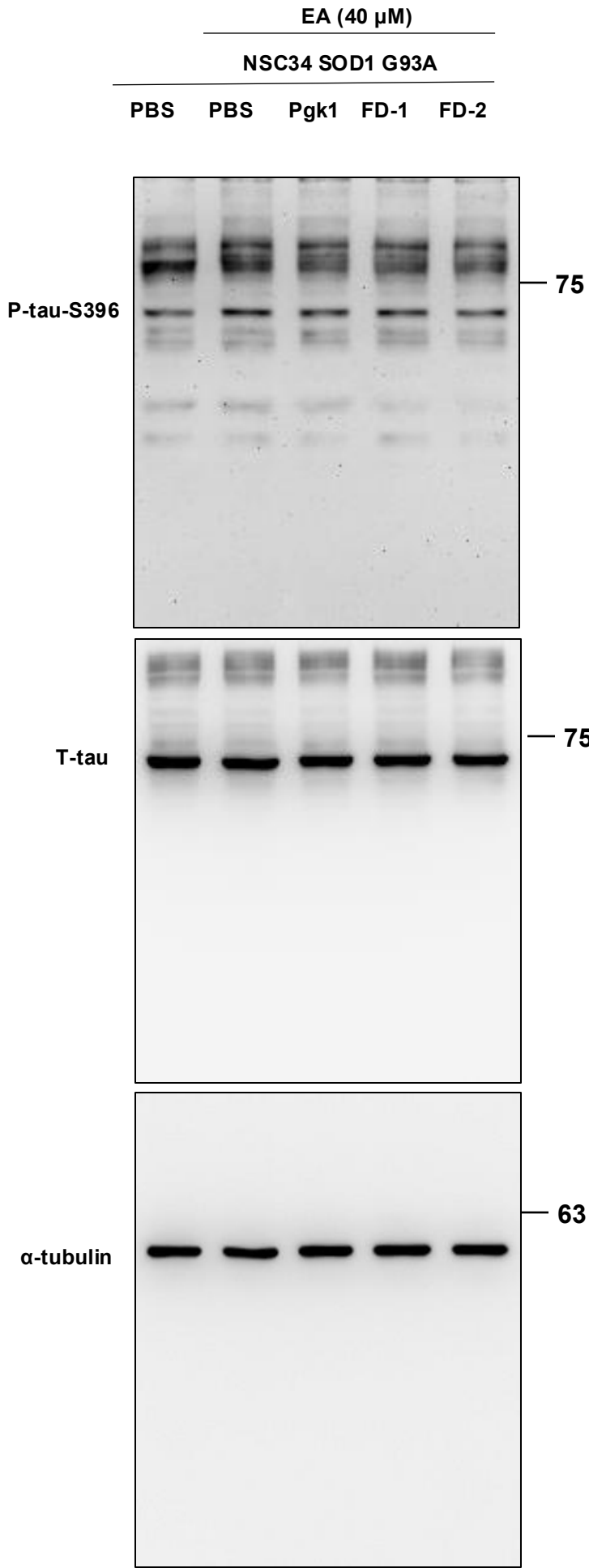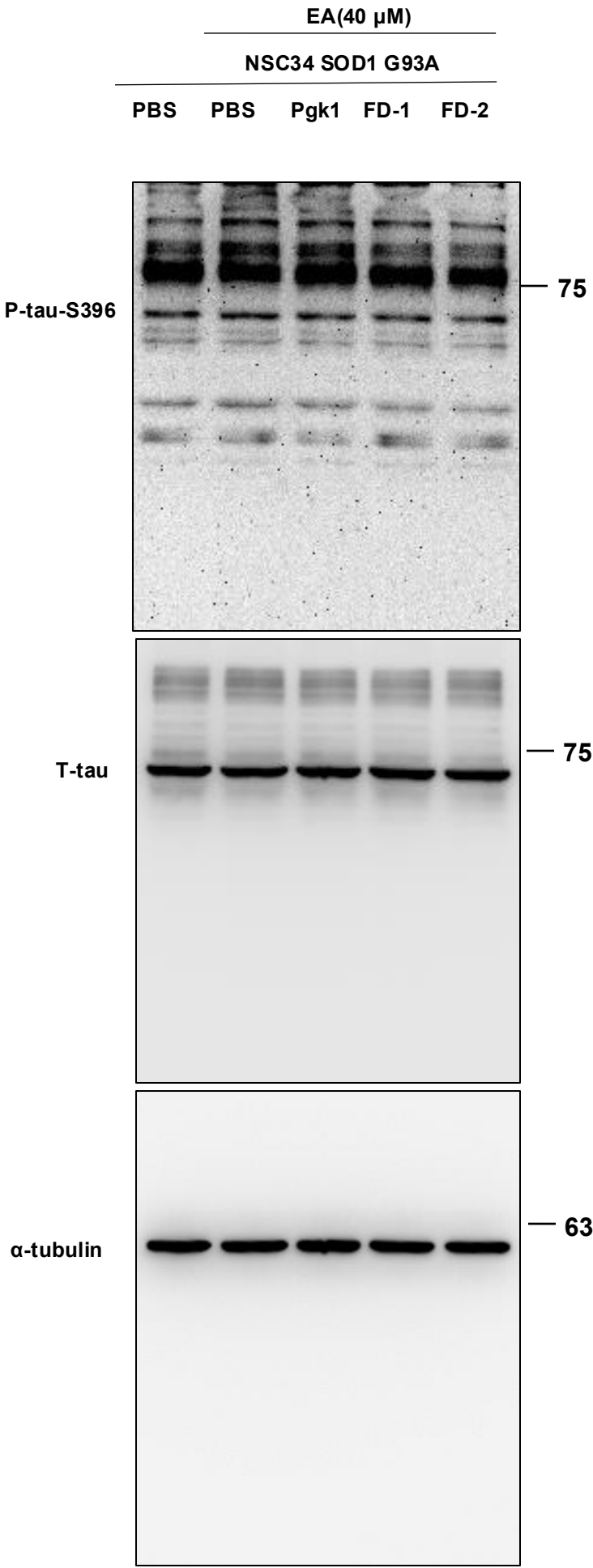

Figure 5g

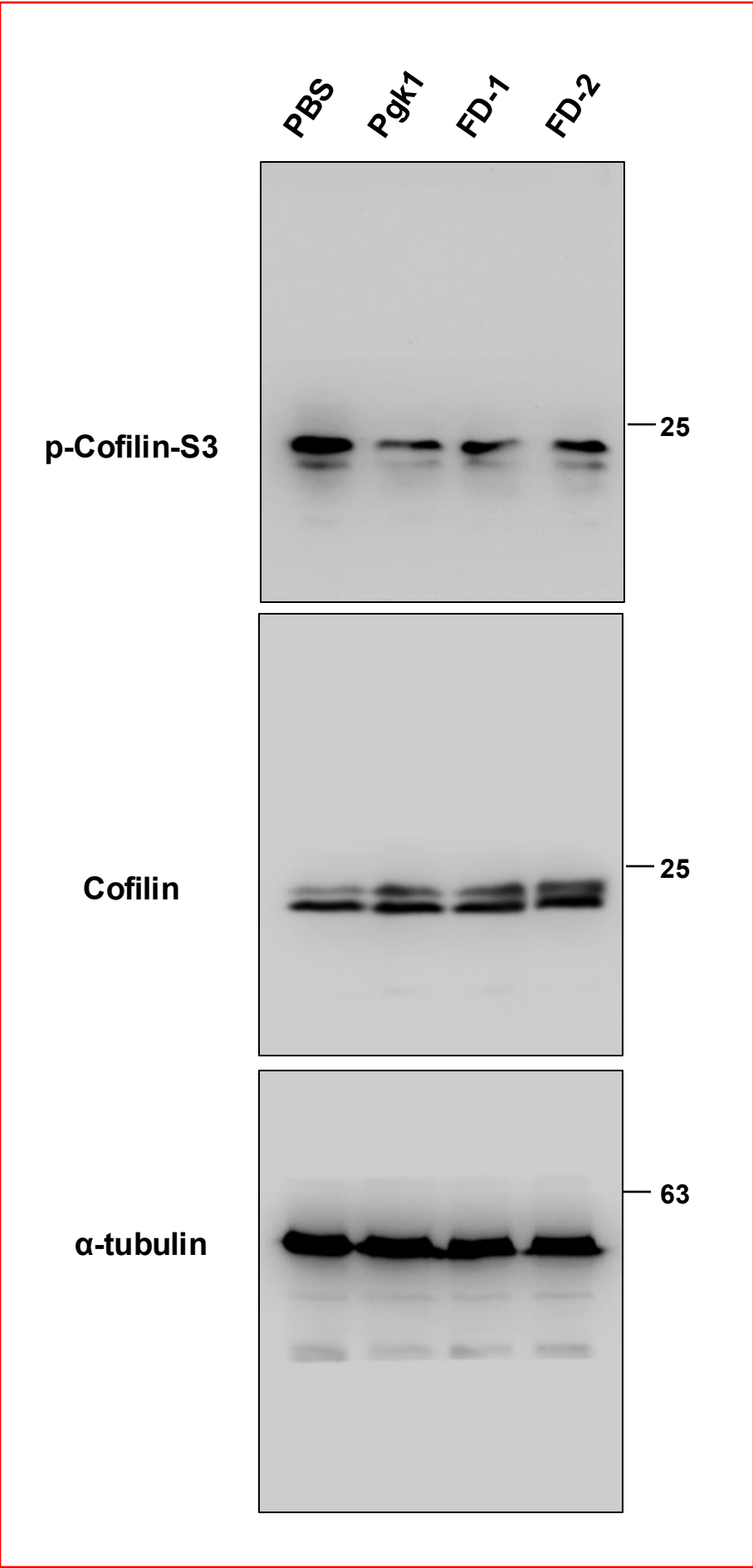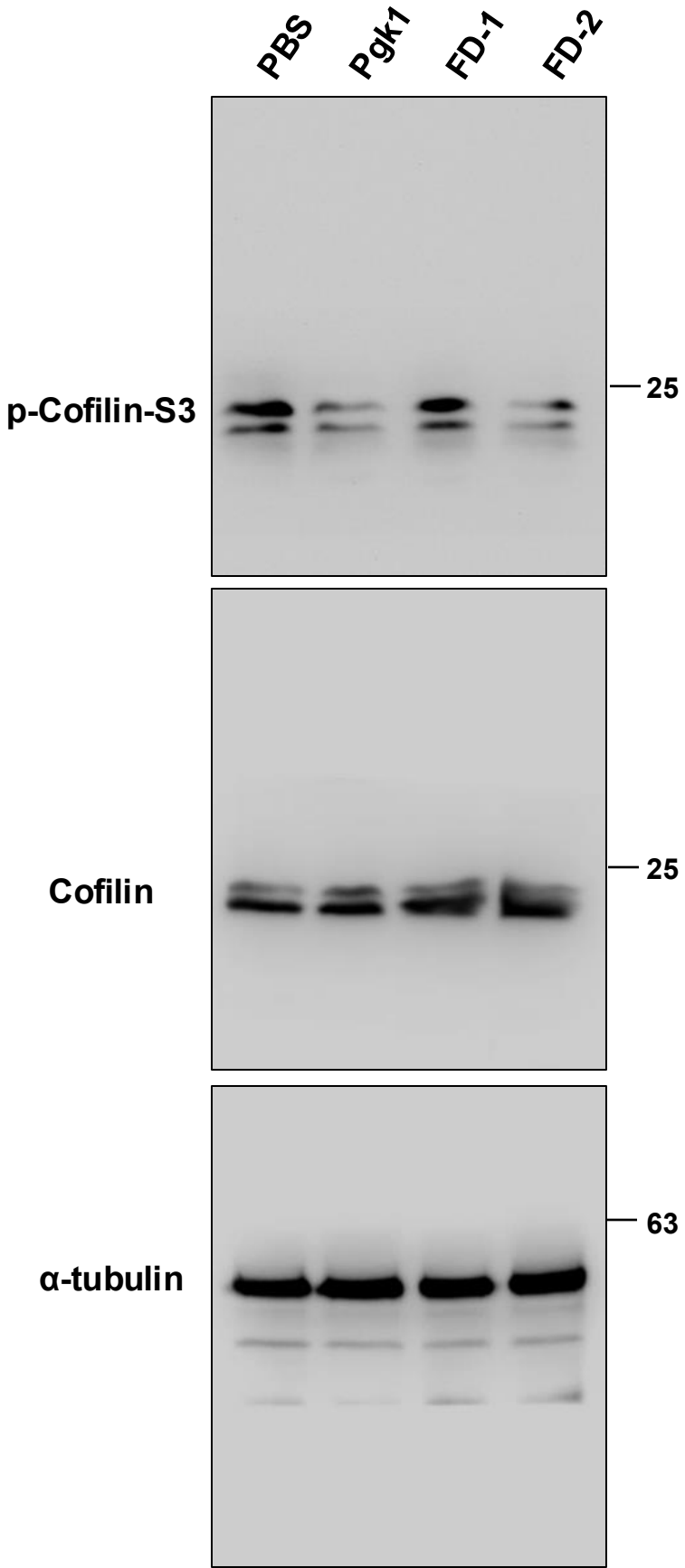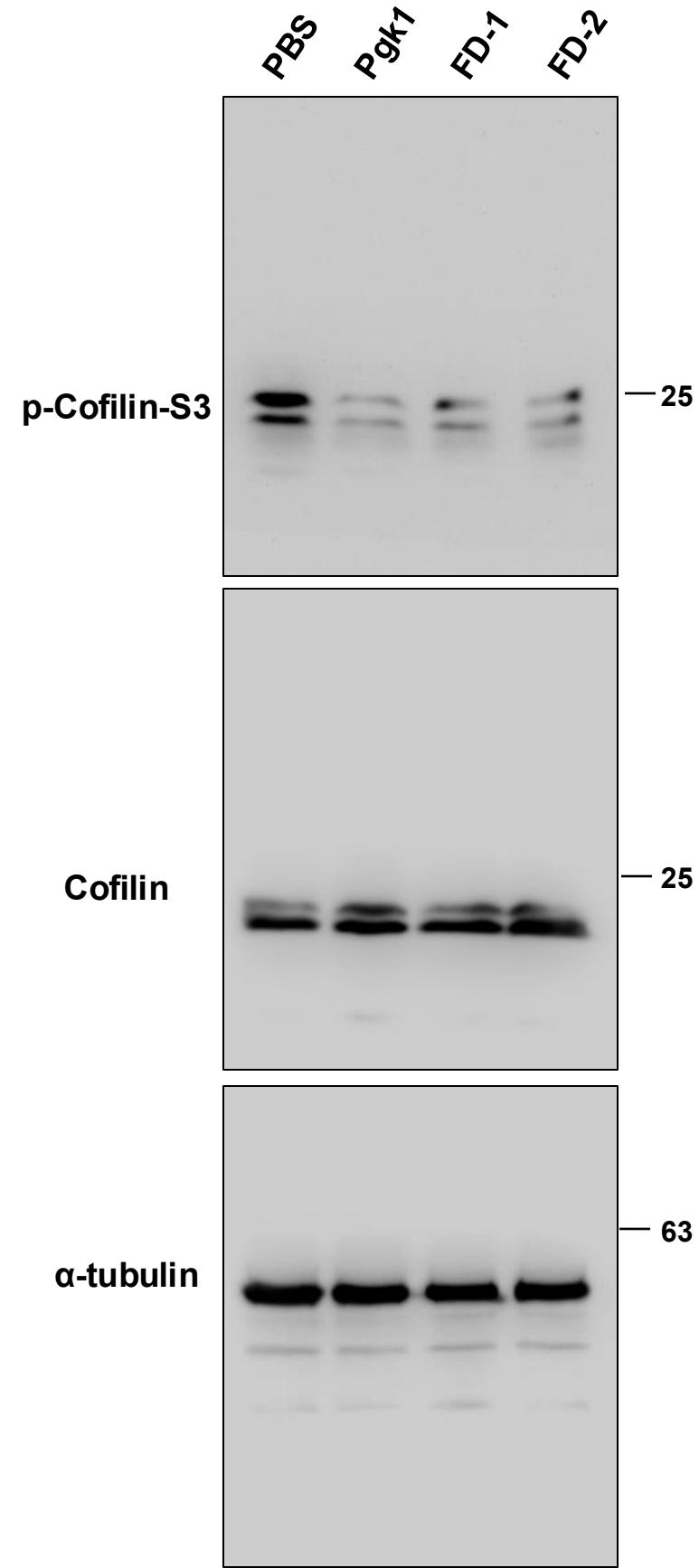

Supplement: Multimedia component 1 [file mmc1.pdf]
